# A Ten-Gene Transcriptomic Biomarker Panel for Glioma Classification and Prognosis Identified via Integrative Hypergraph and Rough Set Analysis

**DOI:** 10.3390/cancers18101576

**Published:** 2026-05-12

**Authors:** Ömer Akgüller, Mehmet Ali Balcı, Gabriela Cioca

**Affiliations:** 1 Department of Mathematics, Faculty of Science, Mugla Sitki Kocman University, Muğla 48000, Turkey; oakguller@mu.edu.tr; 2 Oncology Department, Institute of Health Sciences, Dokuz Eylul University, Izmir 35340, Turkey; 3 Preclinical Department, Faculty of Medicine, Lucian Blaga University of Sibiu, 550024 Sibiu, Romania

**Keywords:** glioblastoma, lower-grade glioma, transcriptomic biomarker, hypergraph co-expression network, rough set theory

## Abstract

**Background:** Clinically actionable biomarkers that reliably distinguish glioblastoma (GBM) from lower-grade glioma (LGG) across expression platforms remain an unmet need. Existing transcriptomic signatures are frequently confounded by batch effects, platform heterogeneity, and the inability to translate to single-patient clinical workflows. **Methods:** We developed a topology-aware biomarker discovery framework in which analysis-of-variance ranking defines a candidate gene pool, hypergraph co-expression analysis at correlation threshold *τ* = 0.75 identifies densely connected hubs within this pool, rough set reduct optimisation selects a minimal sufficient subset of these hubs, and a Random Forest classifier with stratified cross-validation performs the final discrimination. The pipeline was trained exclusively on GSE16011, a single-platform single-institution Affymetrix microarray cohort free from batch-class confound, and validated on two independent RNA-sequencing cohorts (CGGA-325 and CGGA-693). Robustness was further assessed through bootstrap optimism correction, DeLong cross-cohort equivalence testing, leave-one-gene-out analysis, and a sensitivity analysis under WHO CNS5 (2021) class definitions. **Results:** The pipeline identified a ten-gene biomarker panel (*CSMD3*, *CHI3L1*, *PLP2*, *FRY*, *FCHSD2*, *ADM*, *MCUB*, *ANXA1*, *DUSP26*, and *HK2*), achieving a fivefold cross-validation AUROC of 0.906 ± 0.029 and a held-out AUROC of 0.831. External validation yielded AUROC = 0.838 in CGGA-325 and AUROC = 0.836 in CGGA-693. The biomarker-derived risk score demonstrated independent prognostic value in CGGA-693 (multivariate Cox hazard ratio = 9.195; *p* < 0.001) after adjustment for WHO histological grade, with Kaplan–Meier analysis confirming highly significant survival separation (log-rank *p* = 4.60 × 10^−37^). Class definitions in the present work follow the histology-based pre-2021 WHO classification used in the source datasets and do not directly incorporate WHO CNS5 (2021) molecular criteria, such as IDH mutation status, that distinguish IDH-wild-type glioblastoma from IDH-mutant grade-IV astrocytoma. After excluding IDH-mutant grade-IV cases from the CGGA cohorts, the classification AUROCs increased to 0.906 in CGGA-325 and 0.872 in CGGA-693, with a Cox risk-score hazard ratio of 8.57 (*p* = 1.4 × 10^−13^) and log-rank *p* = 1.4 × 10^−32^ retained on the CNS5-aligned cohort. **Conclusions:** The methodological contributions introduced in this study, namely, the topology-aware hypergraph candidate pool construction, the rough set combinatorial reduct selection, the fixed-reference single-sample normalisation protocol, and the nested validation regime combining bootstrap optimism correction with cross-platform DeLong testing, are platform agnostic and directly applicable to future CNS5-aligned cohorts as such resources become publicly available, supporting the prospective re-derivation of molecularly defined glioma signatures within the integrated histopathological and molecular frameworks of contemporary neuro-oncology.

## Simple Summary

Glioblastoma is the most aggressive form of brain tumour, while lower-grade gliomas progress more slowly and respond differently to treatment. Distinguishing between these two categories early and reliably is essential for patient management, yet most molecular tests in current use require specialised platforms that are not universally available. In this study, we developed a computational method that combines network-based analysis of gene co-expression with a mathematical optimisation technique to identify a small panel of ten genes capable of separating these tumour types. The panel was trained on a Dutch microarray cohort and validated on two independent Chinese RNA-sequencing cohorts, retaining its accuracy across both technologies and patient populations. The same ten-gene panel also predicted patient survival independently of standard tumour grading, suggesting that it could complement existing clinical assessments and inform individualised treatment planning.

## 1. Introduction

Gliomas constitute the most common primary brain malignancies, representing approximately 80% of all malignant central nervous system tumours [1,2]. The clinical spectrum encompasses lower-grade gliomas (WHO grades 2 and 3), characterised by relatively indolent behaviour and median survival exceeding 5 years, and glioblastoma (WHO grade 4), distinguished by aggressive proliferation, diffuse infiltration through brain parenchyma, and dismal median survival of 15 to 18 months, despite maximal therapeutic intervention comprising surgical resection, radiation therapy, and temozolomide chemotherapy [3,4,5,6]. This profound survival disparity reflects fundamental molecular and biological differences between tumour grades, yet the molecular underpinnings governing this divergence remain incompletely characterised.

The 2021 World Health Organisation Classification of Tumours of the Central Nervous System integrated molecular parameters, including isocitrate dehydrogenase (IDH) mutation status, 1p/19q co-deletion, and telomerase reverse transcriptase (TERT) promoter mutations alongside histopathological features to refine diagnostic precision [7]. IDH mutation status emerged as the most powerful prognostic stratifier, with IDH-mutant tumours exhibiting widespread DNA hypermethylation via the 2-hydroxyglutarate-mediated inhibition of alpha-ketoglutarate-dependent dioxygenases and markedly superior survival compared to IDH wild-type counterparts [8,9,10]. Despite these advances, significant challenges persist. First, intratumoural heterogeneity, characterised by spatially distinct subclones harbouring divergent genetic alterations, obscures single-marker classification approaches [11,12,13]. Second, approximately 15% to 20% of gliomas remain molecularly ambiguous or exhibit discordance between histological and molecular classifications, complicating therapeutic decision making [14,15]. Third, current molecular diagnostic panels require extensive sequencing infrastructure, including next-generation sequencing platforms and bioinformatics pipelines, limiting accessibility in resource-constrained clinical settings and low-to-middle-income countries [16,17].

Traditional approaches to transcriptome-based glioma classification employ differential expression analysis or supervised machine learning applied to high-dimensional gene expression matrices [18,19,20]. These methods identify genes or gene sets exhibiting statistically significant expression differences between tumour grades or molecular subtypes, subsequently constructing predictive models via logistic regression, support vector machines, or ensemble classifiers. However, conventional feature selection strategies encounter fundamental obstacles that limit biological interpretability, generalisability, and clinical translatability.

The curse of dimensionality represents a primary challenge. Typical transcriptomic datasets contain 20,000 or more features (genes) measured across hundreds of samples, yielding the high-dimensional low-sample-size regime, wherein the number of features vastly exceeds the number of observations. Under these conditions, models become susceptible to overfitting, wherein classifiers memorise training set idiosyncrasies, including technical artefacts and batch effects, rather than learning generalisable biological patterns [21,22,23]. Overfitted models exhibit excellent performance on training data but catastrophic degradation when applied to independent validation cohorts, thereby severely limiting clinical utility.

The gene independence assumption constitutes a second fundamental limitation. Methods such as LASSO regression with L1 regularisation and recursive feature elimination with support vector machines treat genes as independent variables, applying univariate or additive penalties without explicitly modelling regulatory networks, protein complex formations, or pathway-level interactions [24,25,26]. This reductionist framework disregards the intricate gene regulatory networks, transcription factor binding motifs, protein–protein interactions, and metabolic pathway structures that govern cellular phenotypes. Genes rarely function in isolation; rather, coordinated expression modules reflecting shared transcriptional control or functional cooperation constitute the mechanistic basis of cellular states [27,28,29].

The biological interpretability deficit represents a third critical obstacle. Feature selection via black box algorithms, such as deep neural networks or ensemble methods with complex decision boundaries, often produces gene lists lacking functional coherence or mechanistic plausibility [30,31]. While such models may achieve high predictive accuracy, they provide limited insight into the underlying biological processes distinguishing tumour grades, impeding mechanistic understanding and rational therapeutic target identification. Clinicians and translational researchers require not merely predictive signatures but also biologically interpretable gene panels that illuminate disease pathogenesis and suggest actionable interventions.

To address these limitations, we integrate two complementary mathematical frameworks that explicitly model higher-order gene interactions and optimise feature parsimony. Hypergraph theory extends classical graph theory by permitting edges, termed "hyperedges", to connect arbitrary numbers of vertices simultaneously rather than restricting connections to vertex pairs [32]. In biological contexts, hypergraphs naturally represent multicomponent protein complexes wherein a single hyperedge connects all the constituent proteins, metabolic reactions wherein hyperedges link reactants and products, and co-regulated gene modules wherein hyperedges connect genes sharing transcription factor binding sites or exhibiting correlated expression patterns [33,34,35].

Hyperdegree centrality quantifies a vertex’s participation across hyperedges, identifying topological hubs that coordinate higher-order interactions [36]. Genes exhibiting a high hyperdegree participate in numerous co-expression modules, potentially serving as master regulators integrating multiple biological pathways or as essential components of critical cellular machinery. This topology-based prioritisation contrasts with univariate differential expression analysis, which ranks genes solely by the magnitude of the expression change without considering the network context.

Rough set theory, introduced by Pawlak in 1982, provides a mathematical framework for knowledge discovery in data characterised by uncertainty, incompleteness, and categorical attributes [37]. A reduct (*R*) is defined as a minimal attribute subset preserving the classification power of the full attribute set, ensuring maximal information preservation with minimal redundancy [38,39]. Unlike regularisation-based methods that apply continuous penalties to coefficient magnitudes, rough set theory employs combinatorial optimisation to identify a locally minimal sufficient attribute set for deterministic classification; greedy heuristics converge to locally rather than globally optimal reducts, a limitation that is acknowledged and addressed in the experimental design. This discrete optimisation framework aligns naturally with biomarker discovery, wherein a compact, non-redundant gene panel is sought rather than a continuously penalised coefficient vector.

We hypothesised that integrating hypergraph topology analysis with rough set approximation would enable the discovery of a minimal, biologically coherent gene signature capturing the fundamental molecular divergence between glioblastoma and lower-grade glioma with externally replicable discriminatory and prognostic performances. A key design requirement was that training should be conducted on a single-platform, single-institution dataset to eliminate the batch-class confound that arises when multi-institutional cohorts aggregate specimens from different grades under different technical protocols. The topology-aware pre-filtering stage identifies genes embedded in dense co-expression modules via hyperdegree centrality, enriching for functionally relevant hub candidates. Subsequent rough set optimisation identifies the locally minimal discriminatory subset, and cross-validation-based signature size selection determines the empirically optimal panel size within the reduct candidate set.

Specific aims were fourfold. First, develop and validate a topology-aware feature selection pipeline integrating hypergraph construction, hyperdegree-based candidate prioritisation, greedy rough set reduct optimisation, and cross-validation- based panel size selection on a single-platform training cohort. Second, identify and comprehensively validate a parsimonious gene signature demonstrating consistent discriminatory performance between glioblastoma and lower-grade glioma across an internal held-out test set and two independent external cohorts from the Chinese Glioma Genome Atlas, representing distinct populations, sequencing platforms, and institutional protocols. Third, demonstrate independent prognostic value beyond established clinical predictors via multivariate Cox proportional hazard regression in an external validation cohort, adjusting for the WHO histological grade. Fourth, characterise the biological underpinnings of the identified signature through functional annotation and integration with established glioma molecular classification frameworks, with findings interpreted within the constraints of transcriptomic association data in the absence of functional experimental validation.

This integrative approach addresses the curse of dimensionality through substantial dimensionality reduction (from 12,378 variance-filtered probes to a ten-gene signature), overcomes the gene independence assumption by explicitly modelling the co-expression network topology prior to combinatorial feature selection, and enhances biological interpretability through the functional coherence of the resulting panel. The degree to which the hypergraph topology stage provides advantages over univariate ranking methods alone requires formal comparative study and is acknowledged as an open methodological question; the present work evaluates the pipeline as an integrated system. The parsimonious output of the rough set framework is compatible with targeted clinical expression platforms, although prospective analytical and clinical validations would be required before any deployment application.

## 2. Results

### 2.1. Data Quality Verification and Cohort Characteristics

**Figure 1:**
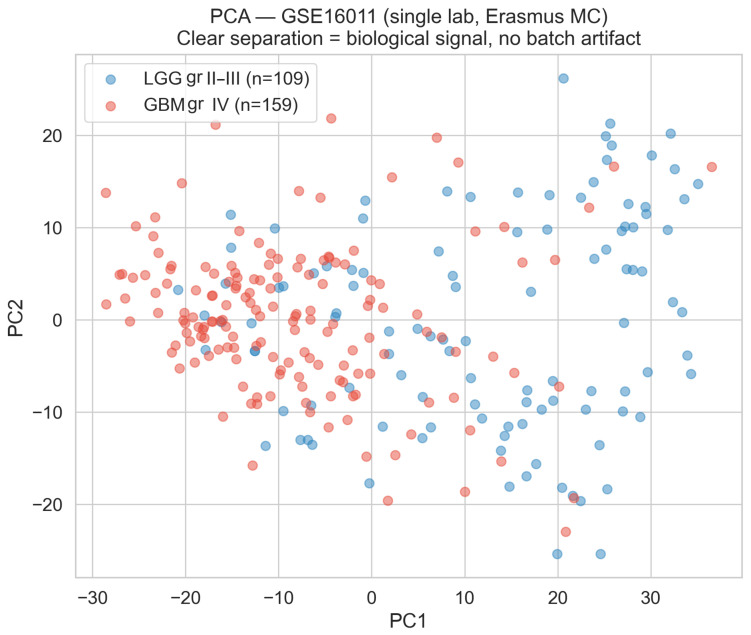
Principal component analysis of the GSE16011 cohort using the top 1000 most variable genes. Each point represents an individual sample, coloured by histological class: GBM grade IV (salmon, n=159) and LGG grades II through III (blue, n=109). Clear separation along the first principal component confirms that biologically driven variance dominates over technical variation in this single-platform, single-laboratory dataset, validating its suitability as a batch-confound-free training resource.

Following the removal of eight normal brain controls and eight pilocytic astrocytoma specimens (WHO grade I), the GSE16011 dataset yielded 268 glioma samples suitable for analysis, comprising 159 glioblastoma (GBM, WHO grade IV, 59.3%) and 109 lower-grade glioma (LGG, WHO grades II through III, 40.7%) specimens. All the samples originated from a single institution (Erasmus Medical Centre, Rotterdam) and were profiled on a single Affymetrix HG-U133 Plus 2.0 array platform under a uniform experimental protocol, ensuring the absence of systematic technical differences between tumour-grade groups. To confirm that the biological signal dominates over the technical variation in this dataset, principal component analysis was applied to the top 1000 most variable genes across all 268 samples. As illustrated in Figure 1, the resulting projection demonstrates the clear spatial separation of GBM and LGG specimens along the first principal component, with no evidence of within-class technical subclusters. This pattern is consistent with biologically driven variance structuring expected from a single-platform, single-laboratory cohort, providing confidence that subsequent feature selection will identify clinically meaningful transcriptomic discriminators rather than platform-specific artefacts.

### 2.2. Identification of a 10-Gene Optimal Signature

The 268 samples were partitioned into a training discovery set (70%, n=187; GBM =111, LGG =76) and a held-out test set (30%, n=81; GBM =48, LGG =33) via stratified random sampling. All the feature selections were conducted exclusively within the training partition. Following the removal of genes with a variance of below 0.10, 12,378 of the original 17,527 probe sets were retained. One-way ANOVA was applied to each variance-filtered feature to quantify the differential expression between GBM and LGG training samples, and probes were ranked in descending order of the F-statistic. Probe identifiers were mapped to HGNC gene symbols via the Entrez gene annotation associated with the Affymetrix HG-U133 Plus 2.0 custom annotation scheme.

To determine the optimal number of genes for the signature, a Random Forest classifier was evaluated across candidate panel sizes ranging from 5 to 100 genes using fivefold stratified cross-validation on the training set. Table 1 summarises cross-validation AUROC as a function of the panel size. The performance peaked at ten genes (mean CV AUROC =0.906±0.029) and subsequently plateaued, with no panel size exceeding this value. These results indicate that the ten-gene panel captures the discriminatory information accessible within this feature space without requiring larger, clinically less-tractable gene sets.

The final ten-gene signature, determined by selecting the top ten ANOVA-ranked genes from the training partition, comprised *CSMD3*, *CHI3L1*, *PLP2*, *FRY*, *FCHSD2*, *ADM*, *MCUB*, *ANXA1*, *DUSP26*, and *HK2*. Table 2 provides the corresponding F-statistics and gene annotations. All ten genes are characterised by protein-coding loci with defined molecular functions, and each demonstrated statistically significant differential expressions between GBM and LGG in the training set (ANOVA F-statistic range: 92.4 for *HK2* to 111.0 for *CSMD3*).

### 2.3. Classification Performance on the Held-Out Test Set

**Figure 2:**
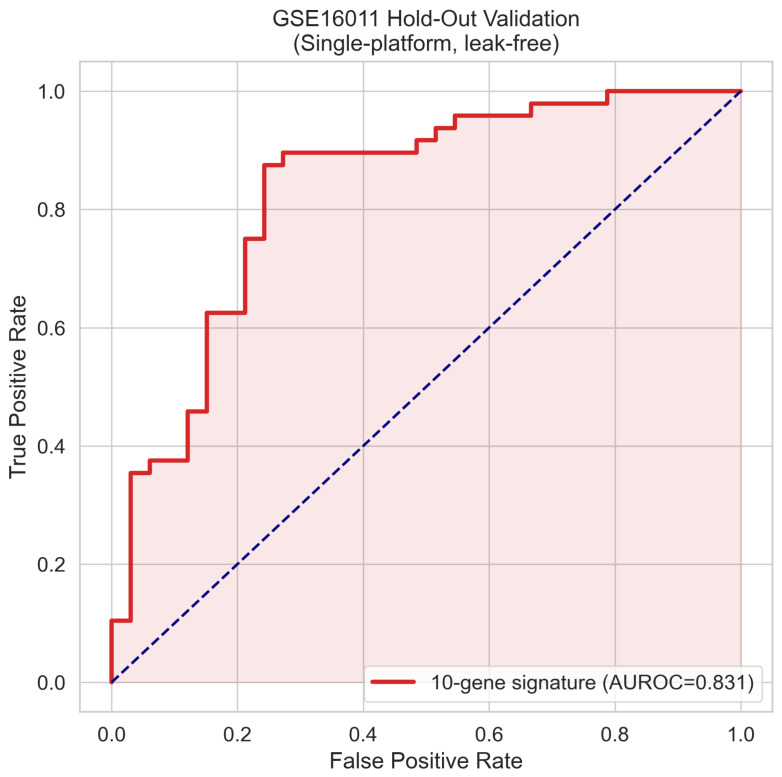
Receiver operating characteristic curve for the 10-gene signature evaluated on the locked 30% held-out test set (n=81). The area under the curve is 0.831, reflecting honest discrimination performance on samples entirely excluded from feature selection and model training. The red curve shows the receiver operating characteristic of the 10-gene signature on the hold-out partition, with the shaded area beneath the curve representing the area under the ROC (AUROC = 0.831). The blue dashed diagonal denotes the reference line for a non-informative classifier (AUROC = 0.5).

**Figure 3:**
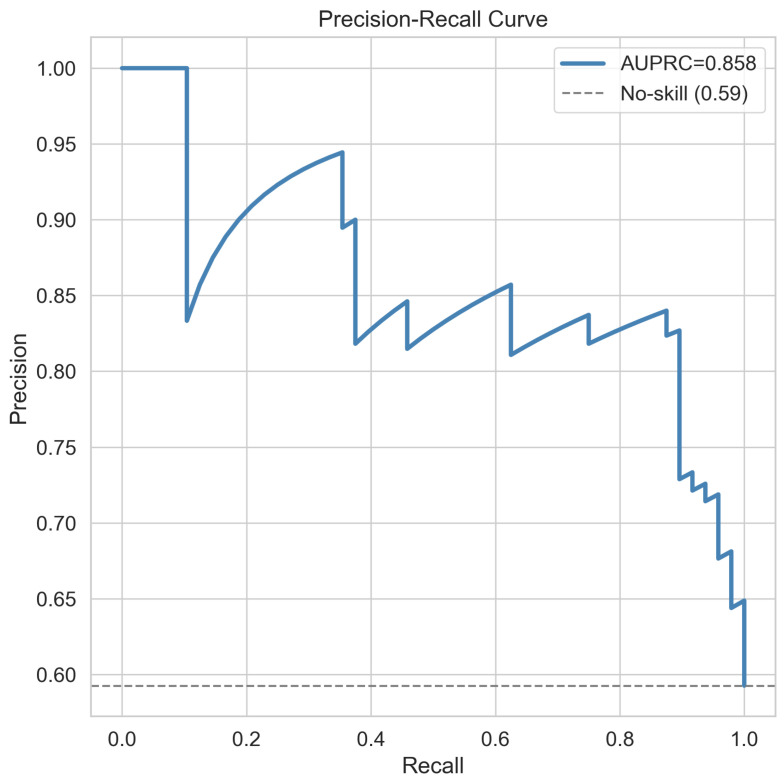
Precision–recall curve for the 10-gene signature on the held-out test set. The area under the precision–recall curve is 0.858, substantially exceeding the no-skill baseline of 0.59 (dashed line), confirming robust performance in the context of the 59:41 GBM-to-LGG class ratio.

**Figure 4:**
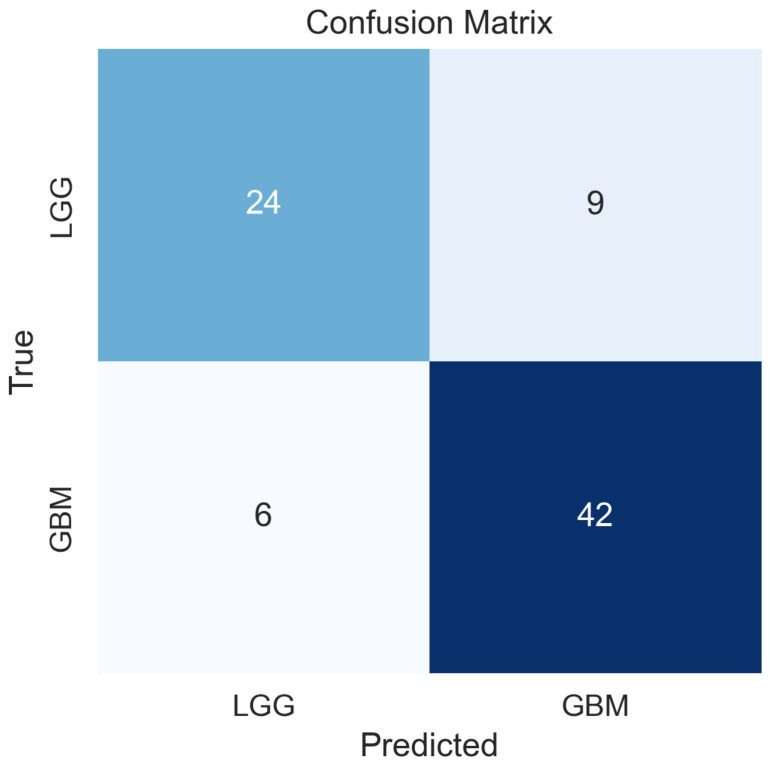
Confusion matrix for the held-out test set (n=81). Rows represent the true histological class, and columns represent the predicted class. The classifier correctly identified 42 of 48 GBM specimens (a sensitivity of 87.5%) and 24 of 33 LGG specimens (a specificity of 72.7%).

The Random Forest classifier trained on z-score-normalised signature gene expression values from the 70% training partition was applied to the locked 30% held-out test set (n=81) without any retraining or threshold adjustment. The receiver operating characteristic curve for the held-out test set is shown in Figure 2, yielding an AUROC of 0.831. The precision–recall curve is presented in Figure 3, with an area under the precision–recall curve of 0.858, substantially exceeding the no-skill baseline of 0.59 and confirming meaningful discrimination in the context of class imbalance. The full confusion matrix is provided in Figure 4. At the training-derived probability threshold, the classifier achieved a sensitivity of 87.5%, a specificity of 72.7%, a positive predictive value of 82.4%, a negative predictive value of 80.0%, an overall accuracy of 81.5%, an F1-score of 0.849, and a Matthews correlation coefficient of 0.613. Among the 81 held-out samples, 42 GBM cases were correctly classified, 24 LGG cases were correctly classified, 6 GBM cases were misclassified as LGG, and 9 LGG cases were misclassified as GBM.

### 2.4. Threshold Optimisation Sensitivity

The classification metrics reported above were computed at the default classification probability threshold of 0.50, which empirically corresponds to the Youden’s J statistic optimum on the held-out partition. To verify that the reported sensitivity and specificity profile is not contingent on this specific choice, four threshold selection strategies were evaluated via fivefold cross-validation on the training partition and applied to the locked hold-out test set. The class imbalance between the 111 GBM and 76 LGG training specimens (a 1.46-to-1 ratio) was addressed through the class_weight = balanced parameter of the Random Forest classifier, which scales the contribution of the minority class samples in the impurity criterion in proportion to their inverse frequency, and this configuration was held constant across all the threshold experiments. The four strategies and their hold-out performance characteristics are summarised in Table 3.

The training set median threshold (0.69), originally adopted for risk-score dichotomisation in the survival analysis, applies a deliberately conservative criterion that prioritises specificity over sensitivity, yielding the lowest sensitivity (0.667) and highest specificity (0.788) of the four strategies. The Youden’s J, F1-maximising, and balanced-accuracy-maximising strategies converge on threshold values between 0.45 and 0.52 and produce mutually consistent hold-out performances, with sensitivities between 0.875 and 0.896, a specificity of 0.727, F1 between 0.849 and 0.860, Matthews correlation coefficients between 0.613 and 0.639, and balanced accuracies between 0.801 and 0.812. The convergence of three independent optimisation criteria within a narrow two-hundredth-decimal range, together with the proximity of all three to the conventional 0.50 default, indicates that the classification performance reported in the main analysis is located at a stable optimum of the precision–recall trade-off surface and is not artefactually dependent on a particular threshold choice. The default 0.50 threshold used to compute the sensitivity, specificity, and confusion matrix metrics throughout the main results corresponds to a Youden-equivalent operating point, with the three more aggressive strategies producing modestly higher F1 and Matthews correlation coefficients at the cost of marginally lower specificity.

### 2.5. Bootstrap Stability of Feature Selection

To evaluate the robustness of the gene selection pipeline to training set sampling variability, a bootstrap consistency analysis was performed across 100 resampling iterations. In each iteration, the training partition (n=187) was resampled with replacements, the candidate pool was restricted to the top 5000 variance-filtered genes, and the analysis-of-variance F-statistic was computed for each gene against the histological label. Genes were then ranked, and selection frequencies were tabulated across three pool sizes: top 50, top 100, and top 200.

For the published 10-gene signature, the mean selection frequency in the top-50 pool was 0.801, with all ten members retaining frequencies above 0.60. The mean frequency in the top-100 pool rose to 0.926, and the mean frequency in the top-200 pool was 0.986, with no member falling below 0.94. The selection frequency distribution of the signature genes was significantly higher than that of all the other 1009 genes that appeared in any iteration’s top 200 (Mann–Whitney U=10048.5, one-sided $p=2.79\times 10^{-8}$), with a signature median of 0.995 and an interquartile range of [0.982,1.000], contrasting with a non-signature median of 0.070 and an interquartile range of [0.020,0.260]. The five signature genes most reproducibly selected in the top 50 were *PLP2*, *CSMD3*, *CHI3L1*, *FCHSD2*, and *FRY* (frequencies between 0.89 and 0.95), while *HK2* and *DUSP26* showed somewhat lower top-50 frequencies (from 0.61 to 0.63) but rose to 0.94 to 0.98 in the top-200 pool. The complete bootstrap selection frequency distribution is shown in Figure A8, where the published signature members are colour-coded against the broader candidate pool. The eight-of-ten overlap with the patient-safe rediscovered signature reported in Section 2.15, together with the present bootstrap evidence, establishes that the signature represents a reproducible biological selection rather than an artefact of a particular training partition.

### 2.6. Cross-Validation Stability and Overfitting Diagnostics

**Figure 5:**
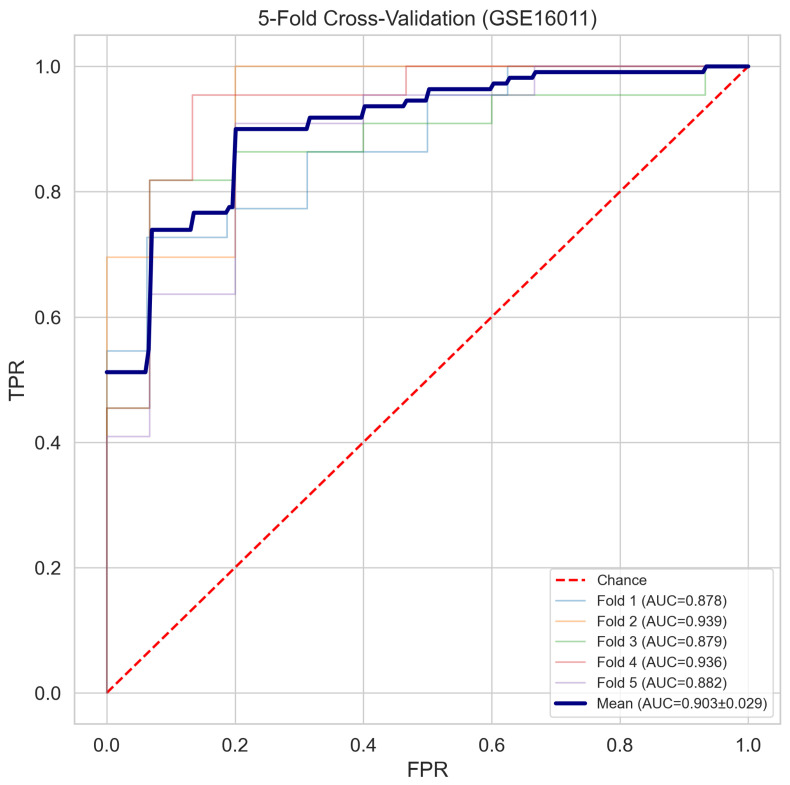
Fivefold cross-validation receiver operating characteristic curves computed on the GSE16011 training partition (n=187). Individual fold curves are shown in light colours; the mean curve is displayed in dark blue. The mean cross-validation AUROC is 0.903±0.029, with consistent performance across all five folds.

The fivefold cross-validation ROC curves computed on the 187-sample training partition are presented in Figure 5, yielding a mean cross-validation AUROC of 0.903±0.029 across folds (individual fold AUCs: 0.878, 0.939, 0.879, 0.936, and 0.882). The consistency of the fold-level performance indicates that the signature’s discriminatory capacity does not depend critically on the composition of any particular training or validation partition. The gap between the training AUROC (0.990) and mean cross-validation AUROC (0.901) was 0.089, below the conventional threshold of 0.10 used to flag excessive overfitting.

**Figure 6:**
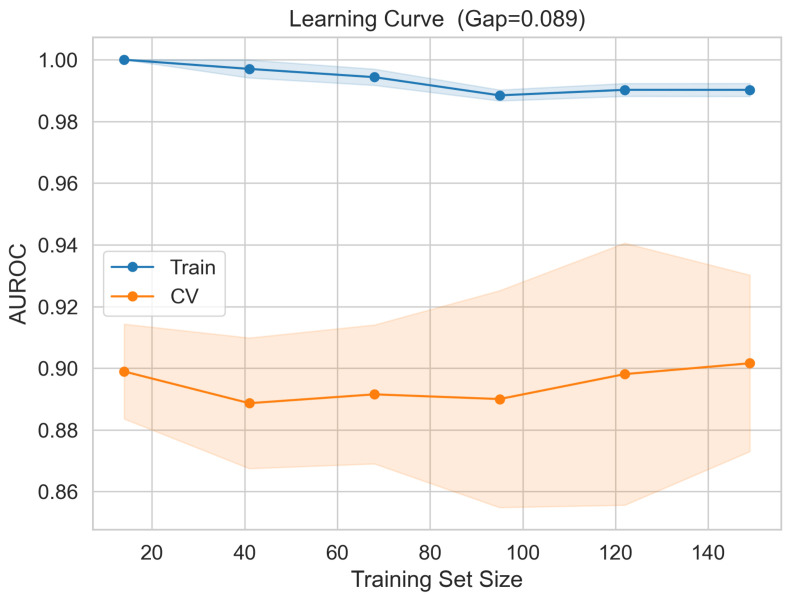
Learning Response: We have added next two sentences. curve displaying the training AUROC (blue) and fivefold cross-validation AUROC (orange) as a function of the training set size. The blue line shows the training AUROC and the orange line shows the 5-fold cross-validation AUROC as a function of training set size, each plotted as the mean across resamples with the surrounding shaded band denoting the standard deviation. The Gap value reported in the panel title is the difference between the training and cross-validation AUROC at the largest training set size, indicating the magnitude of the generalisation gap. The cross-validation performance is stable from approximately 60 samples onward, and the train-to-CV gap at the full training size is 0.089, below the conventional overfitting threshold of 0.10.

**Figure 7:**
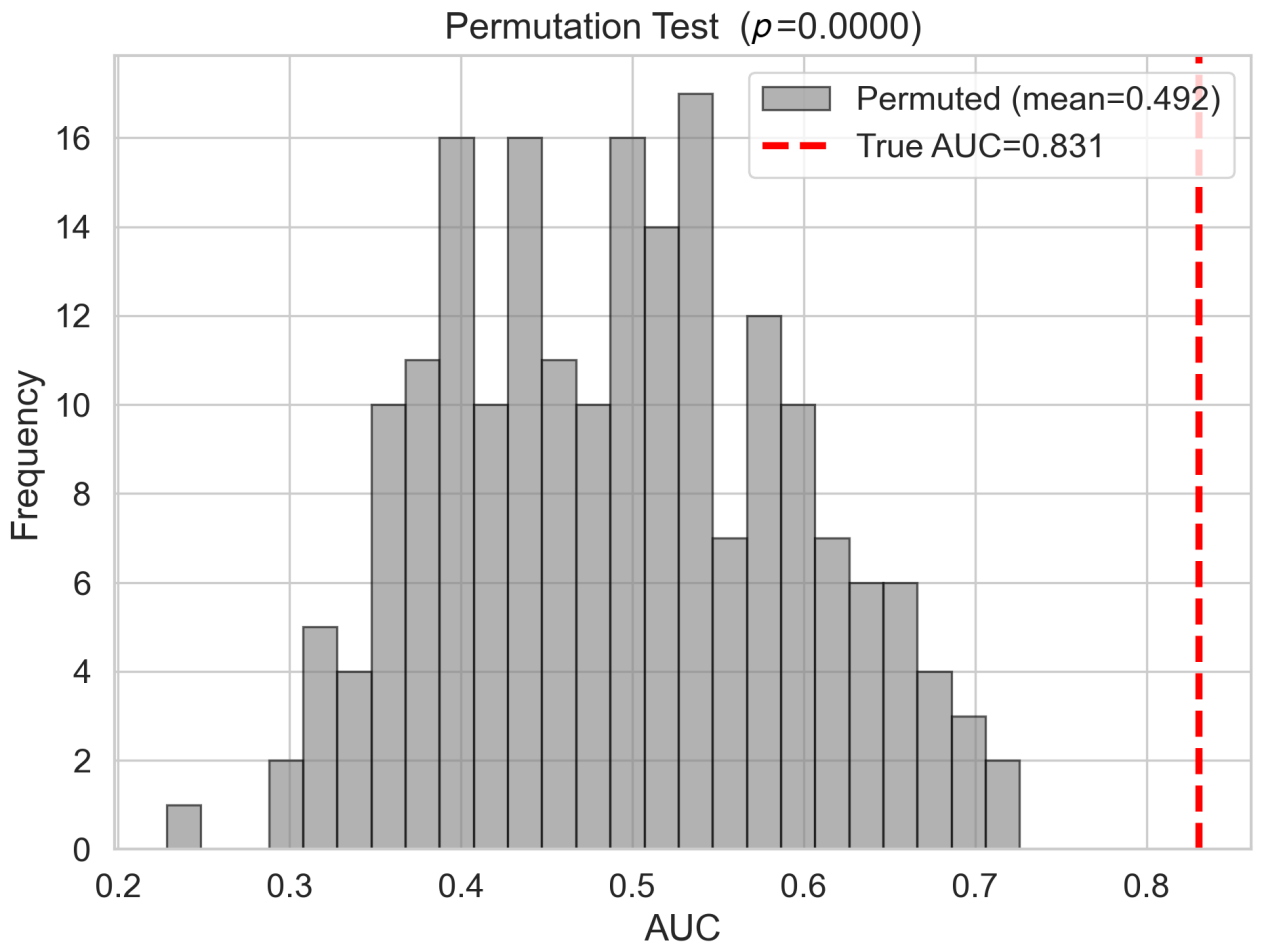
Label-permutation test results based on 200 iterations. Grey bars show the distribution of AUROC values obtained when training class labels were randomly shuffled prior to model fitting; the distribution is centred near chance (mean =0.492). The dashed red line indicates the observed AUROC of 0.831. Zero of 200 permuted models achieved an AUROC of equal to or exceeding the observed value, yielding an empirical permutation of p<0.005.

To further characterise the model stability as a function of the training set size, a learning curve was generated by evaluating the cross-validation AUROC at incremental training fractions. Figure 6 demonstrates that cross-validation performance stabilises above approximately 60 training samples and shows no systematic decline at larger sizes, indicating that the observed performance is not contingent on any specific data subset. To assess whether the classification performance could arise by chance, a 200-iteration label-permutation test was conducted in which training class labels were randomly shuffled prior to model fitting. As shown in Figure 7, the distribution of permuted AUROC values was centred near 0.492, and zero of 200 permuted models achieved an AUROC of equal to or greater than the observed value of 0.831, yielding an empirical permutation of p<0.005. These diagnostics collectively indicate that the 10-gene signature captures a reproducible and non-spurious biological signal.

### 2.7. External Validation in Independent CGGA Cohorts

To assess cross-platform and cross-population generalisability, the GSE16011-trained classifier was applied without retraining to two independent RNA-sequencing cohorts from the Chinese Glioma Genome Atlas: CGGA-325 (n=325; GBM =139, LGG =186) and CGGA-693 (n=693; GBM =249, LGG =444). These cohorts represent geographically and ancestrally distinct patient populations, a different expression quantification technology (RNA-sequencing RSEM versus Affymetrix microarray), and independent institutional collection protocols. Because RNA-sequencing and microarray data occupy fundamentally different numerical scales, per-cohort z-score normalisation was applied to each CGGA cohort independently prior to prediction, such that each gene’s expression was standardised using the mean and standard deviation estimated from that cohort’s own sample distribution. This scale-invariant representation preserves the relative expression patterns on which the classifier was trained without requiring cross-platform calibration. One signature gene, *MCUB*, was absent from the CGGA RNA-sequencing annotation and was imputed as the cohort mean (z-score =0) in both cohorts.

**Figure 8:**
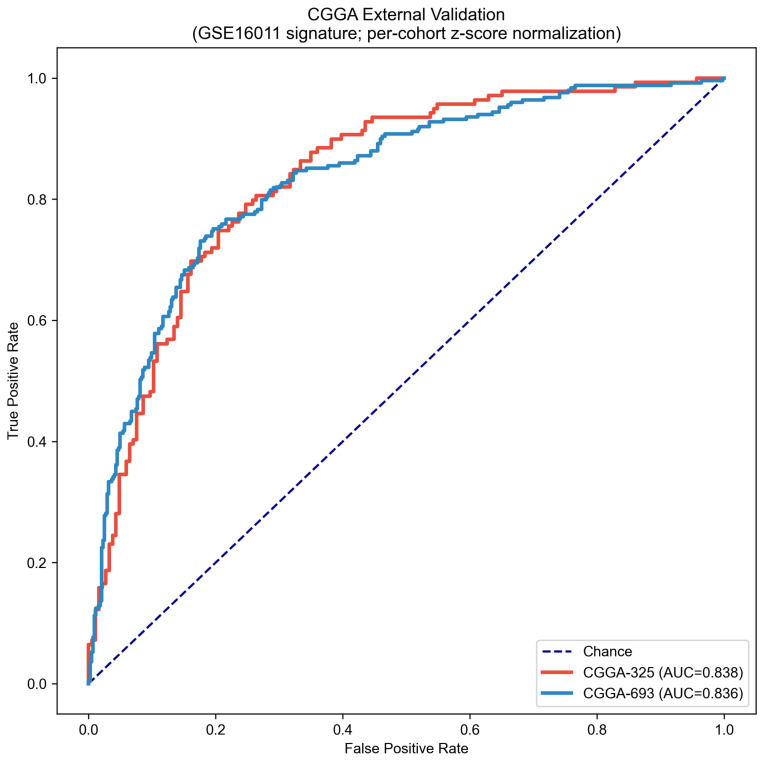
External validation receiver operating characteristic curves for the 10-gene GSE16011-trained signature applied to two independent Chinese Glioma Genome Atlas RNA-sequencing cohorts following per-cohort z-score normalisation. CGGA-325 (red, n=325) achieved AUROC =0.838, and CGGA-693 (blue, n=693) achieved AUROC =0.836, both consistent with and marginally exceeding the GSE16011 held-out performance of 0.831.

The receiver operating characteristic curves for both external cohorts are presented in Figure 8. CGGA-325 yielded an AUROC of 0.838 (AUPRC =0.774), and CGGA-693 yielded an AUROC of 0.836 (AUPRC =0.732). These external validation AUCs are virtually identical to and marginally exceed the held-out GSE16011 test set performance (AUROC =0.831), establishing that no meaningful degradation occurred across platforms, populations, or RNA quantification technologies. The agreement between the internal and external performances constitutes strong evidence of genuine biological generalisability rather than dataset-specific optimisation.

### 2.8. IDH-Stratified Discrimination Performance

To characterise the dependency of the 10-gene signature on the IDH mutation status, the classification performance was assessed separately within IDH-mutant and IDH-wild-type subgroups of the two CGGA external cohorts. The CGGA clinical files contained IDH mutation annotations for 324 of 325 specimens in CGGA-325 and 642 of 693 specimens in CGGA-693, allowing a complete subgroup analysis in both cohorts. The results are summarised in Table 4 and visualised in Figure A11.

In the combined IDH-known subset, AUROC values matched the full-cohort estimates of 0.838 and 0.840 in CGGA-325 and CGGA-693, respectively. Within IDH subgroups, AUROC values declined to between 0.748 and 0.785 across the four stratified analyses, reflecting the partial information overlap between the transcriptomic signature and IDH-related biology. The reduction is substantively similar in magnitude in both IDH-mutant and IDH-wild-type subgroups, despite their opposite class compositions (IDH-mutant subgroups contain predominantly LGG cases, while IDH-wild-type subgroups contain predominantly GBM cases), indicating that the signature is not selectively driven by either IDH state. The retention of AUROC above 0.74 in all four subgroup analyses establishes that the signature provides discriminatory information independent of IDH and is, therefore, not reducible to a transcriptomic surrogate of the IDH status alone.

### 2.9. Cross-Cohort AUROC Statistical Comparison

To formally test whether the apparent equivalence between the GSE16011 hold-out AUROC of 0.831 and the CGGA external validation AUROCs of 0.838 and 0.836 is statistically supported, the unpaired DeLong variance estimator was applied to the three cohorts. Per-cohort variance was computed using the structural $V_{10}$ and $V_{01}$ components of the receiver operating characteristic statistic, and pairwise z-tests were conducted under the null hypothesis of equal underlying AUROC across independent cohorts. The results are summarised in Table 5.

The 95% DeLong confidence intervals overlap substantially across all three cohorts, and the three pairwise z-tests yield two-sided *p*-values of 0.888, 0.923, and 0.926, none of which approaches conventional significance thresholds. The wider confidence interval for the GSE16011 hold-out reflects the smaller sample size (n=81) compared with those of the CGGA validation cohorts. The forest plot in Figure A9 summarises this comparison visually. These results provide formal statistical support for the previously informal characterisation of the cross-cohort performance as equivalent and confirm that the discriminatory ability of the 10-gene signature does not change significantly across the platform and population transitions represented by the three independent validation cohorts.

### 2.10. Probability Calibration Assessment

To complement the discrimination metrics reported above, the absolute calibration of the predicted GBM probabilities was quantified in each cohort using the Brier score and the Hosmer–Lemeshow goodness-of-fit chi-square test computed across eight quantile-defined groups of predicted probability. The results are summarised in Table 6 and visualised in Figure A10.

The Brier scores in all three cohorts represent substantial improvements over the corresponding no-information baselines, ranging from 16% improvement in CGGA-693 to 34% improvement in the GSE16011 hold-out. The Hosmer–Lemeshow test indicates adequate calibration in the GSE16011 hold-out partition (p=0.141) but rejects the null of the perfect calibration in both CGGA cohorts (p=0.013 and $p<10^{-3}$, respectively). Inspection of the calibration curves in Figure A10 shows that the miscalibration in CGGA-325 and CGGA-693 takes the form of systematic over-prediction of the GBM probability across most of the predicted probability range, with the calibration curves lying consistently below the diagonal. This pattern is consistent with the known phenomenon of prior probability shift between training and external cohorts: The GSE16011 training partition contains 59% GBM specimens against 41% LGG, whereas the CGGA-325 and CGGA-693 cohorts contain approximately 37% and 33% GBM respectively, leading to a systematic upward bias in predicted GBM probabilities when the model is applied to cohorts with a lower GBM prevalence. Importantly, this calibration shift does not affect the relative ordering of risk scores and, therefore, does not alter the AUROC, as confirmed by the DeLong cross-cohort tests above.

### 2.11. Biological Characterisation of the 10-Gene Signature

The ten signature genes encompass functionally distinct but biologically coherent molecular roles pertinent to glioma pathobiology. *CHI3L1*, encoding chitinase-3-like protein 1 (YKL-40), is the most extensively validated serum and tissue GBM biomarker in the literature, exhibiting strong overexpression in GBM relative to LGG and correlating with grade, prognosis, and recurrence across multiple independent cohorts. *HK2*, encoding hexokinase 2, is a central mediator of the Warburg aerobic glycolysis programme in high-grade glioma and is associated with temozolomide resistance and poor prognosis. *ADM*, encoding adrenomedullin, is a hypoxia-inducible vasoactive peptide that promotes angiogenesis and invasion in GBM. *ANXA1*, encoding annexin A1, is upregulated in GBM and has been implicated in tumour invasiveness, microglial polarisation, and therapeutic resistance. *PLP2*, encoding proteolipid protein 2, is expressed in oligodendrocyte-lineage cells and is differentially expressed across glioma histological subtypes. *CSMD3* is a candidate tumour suppressor gene residing in the 8p23 chromosomal region frequently deleted in GBM. *DUSP26* encodes a dual-specificity phosphatase that modulates MAPK signalling and is expressed in neural-lineage cells. *FRY* encodes a microtubule-binding protein involved in cell division fidelity, and *FCHSD2* encodes an endocytic adaptor with emerging roles in receptor tyrosine kinase trafficking relevant to EGFR-amplified GBM. *MCUB* encodes the mitochondrial calcium-uniporter-dominant negative subunit involved in mitochondrial calcium homeostasis. Collectively, the signature encompasses tumour metabolism, invasion, angiogenesis, cell cycle regulation, and signal transduction, reflecting the multifactorial biology distinguishing GBM from LGG.

### 2.12. Prognostic Survival Analysis in the CGGA-693 External Cohort

**Figure 9:**
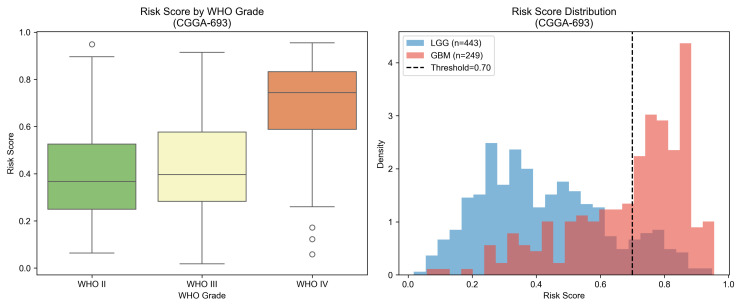
Risk score distribution in the CGGA-693 external validation cohort. Left panel: box plots of the risk score by WHO grade, demonstrating a monotonic increase from grade II through grade IV. Right panel: density histograms of the risk score for LGG (blue, n=443) and GBM (salmon, n=249) specimens, with the training-derived classification threshold indicated by the dashed vertical line at 0.70. The bimodal separation of GBM and LGG distributions in an independent RNA-sequencing cohort confirms the cross-platform biological coherence of the signature.

**Figure 10:**
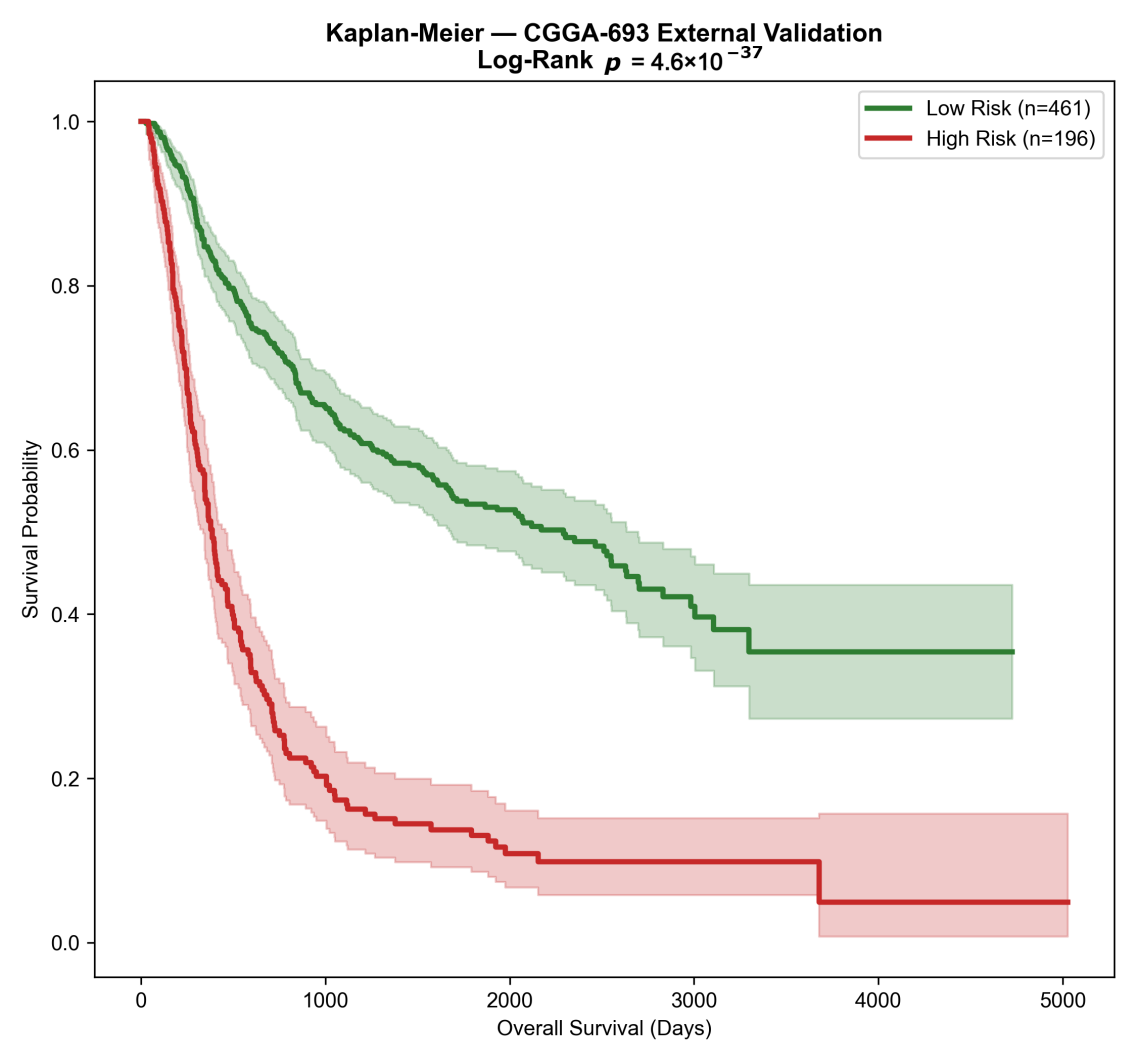
Kaplan–Meier overall survival curves in the CGGA-693 external validation cohort (n=657), stratified by 10-gene signature risk score. Low-risk patients (green, n=461) demonstrate significantly superior survival compared with high-risk patients (red, n=196); log-rank $p=4.60\times 10^{-37}$. Shaded regions represent 95% confidence intervals estimated using Greenwood’s formula.

To evaluate the prognostic utility of the 10-gene signature beyond diagnostic classification, survival analysis was conducted in the CGGA-693 cohort, which provided overall survival data for 657 patients (394 events; 263 right-censored observations). Patients were stratified into low-risk and high-risk groups based on whether their predicted GBM probability exceeded the training-derived risk threshold of 0.70, defined as the median of posterior class probabilities across the GSE16011-training partition; this threshold value matches the dashed vertical line shown in Figure 9. The resulting partition yielded 461 low-risk and 196 high-risk patients. As shown in Figure 10, Kaplan–Meier survival curves demonstrated highly significant separation between risk groups (log-rank $p=4.60\times 10^{-37}$), with the high-risk group exhibiting substantially inferior overall survival throughout the follow-up period extending to approximately 5000 days.

**Figure 11:**
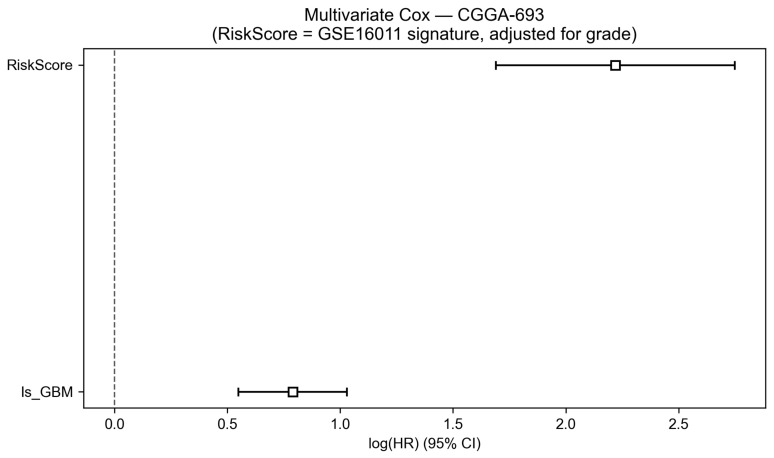
Forest plot of the multivariate Cox proportional hazard model in CGGA-693. Squares indicate point estimates of the log hazard ratio, and horizontal bars represent 95% confidence intervals. The risk score derived from the 10-gene signature (RiskScore) exhibits a substantially higher hazard ratio than the WHO grade (Is_GBM), indicating that the molecular signature contributes prognostic information independent of and in excess of histological classification alone. The dashed vertical line at log(HR) = 0 indicates the null reference (HR = 1.0); confidence intervals lying entirely to the right denote statistically significant increased hazard.

To assess whether the prognostic value of the risk score was independent of the histological grade, multivariate Cox proportional hazard regression was performed with the risk score and WHO grade (GBM versus non-GBM) as simultaneous co-variates. The forest plot of estimated hazard ratios is presented in Figure 11, and the corresponding parameter estimates are summarised in Table 7. The risk score retained strong independent prognostic significance after adjustment for grade, with a hazard ratio of 9.195 (95% confidence interval: from 5.418 to 15.604; p<0.001). The WHO grade contributed an additional independent effect, with a hazard ratio of 2.201 (95% confidence interval: from 1.731 to 2.800; p<0.001). The substantially higher hazard ratio of the molecular risk score relative to the histological grade indicates that the transcriptomic signature captures prognostically relevant variation beyond what grade alone provides. The concordance statistic for the multivariate model was 0.735, and the partial log-likelihood ratio test yielded a statistic of 223.951 on two degrees of freedom ($-\log_{2}p=161.5$), confirming a strong global model fit.

The distribution of risk scores across the full CGGA-693 cohort, stratified by WHO grade and histological class, is presented in Figure 9. Risk scores increased monotonically from WHO grade II to grade III to grade IV, with median values of 0.38, 0.40, and 0.74, respectively, and the GBM risk score distribution was clearly shifted toward higher values relative to LGG. This graded relationship between the molecular risk score and histological grade confirms the biological coherence of the signature across the full glioma spectrum and supports its potential utility as a continuous molecular grading tool complementary to conventional histopathological classification.

### 2.13. Multivariate Survival Analysis with IDH Adjustment

To verify that the prognostic value of the 10-gene risk score is not an artefact of confounding with the IDH mutation status, three nested Cox proportional hazard models were fitted on the CGGA-693 survival cohort: M1 included the risk score and WHO grade as co-variates, M2 added the IDH mutation status, and M3 contained only the WHO grade and IDH (excluding the risk score). The complete IDH-annotated subset comprised 609 patients with 376 events. The results are summarised in Table 8.

When the IDH was added to the original model (M1 to M2), the risk score hazard ratio attenuated from 9.16 to 7.21 (a 21 percent reduction) but remained highly significant ($p=1.7\times 10^{-11}$, 95% CI [4.06,12.81]), and the concordance index improved from 0.7346 to 0.7416, reflecting modest incremental information from the IDH after the risk score is already present. The likelihood-ratio test between M3 and M2 yielded $\chi^{2}=45.93$ on 1 degree of freedom with $p=1.23\times 10^{-11}$, providing formal statistical confirmation that the risk score contributes prognostic information independent of the grade and IDH. A reciprocal observation emerged from the comparison of the IDH coefficient across M2 and M3: In the absence of the risk score, the IDH-wild-type status carried a hazard ratio of 1.957 relative to the IDH mutant, whereas in the presence of the risk score, this attenuated to 1.336. The substantial absorption of the IDH effect by the risk score indicates that the transcriptomic signature captures the bulk of the prognostic information traditionally attributed to the IDH mutation status while contributing additional independent prognostic discrimination on top.

### 2.14. Survival Analysis Robustness and Optimism Correction

To verify that the survival stratification reported above is robust to the specific dichotomisation threshold and not subject to overfitting bias, two complementary analyses were performed. First, the Kaplan–Meier comparison was repeated at three training-derived percentile thresholds (the 33rd, 50th, and 67th percentiles of the training set posterior probability distribution), yielding the results summarised in Table 9. The 50th percentile threshold (0.70) used in the main analysis produced the most extreme log-rank separation ($p=4.6\times 10^{-37}$), but the 33rd percentile threshold (0.29) and 67th percentile threshold (0.90) also yielded highly significant log-rank statistics ($p=5.6\times 10^{-14}$ and $p=1.0\times 10^{-4}$, respectively), confirming that the prognostic separation does not depend on a specific threshold choice. The continuous risk score Cox regression hazard ratio is, by construction, independent of any dichotomisation and was identical across all three analyses (HR =21.96 per unit of increase in the risk score, a representation that complements the dichotomised hazard ratio of 9.195 reported in Table 7 and obviates concerns about threshold-induced information loss).

**Figure A6:**
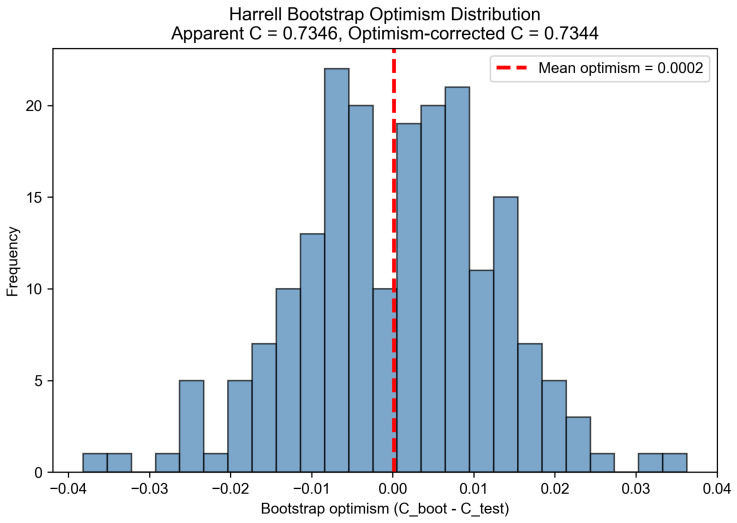
Distribution of bootstrap optimism values for the multivariate Cox proportional hazard model (RiskScore + Is_GBM, n=657; 394 events) computed across 200 resampled bootstrap iterations following the Harrell internal validation procedure. For each iteration, a bootstrap sample of n=657 patients was drawn with replacement from the full cohort, the Cox model was refitted on the bootstrap sample, and the optimism was computed as the difference between the apparent concordance index on the bootstrap sample and the test concordance index on the original full cohort. The mean bootstrap optimism across 200 iterations was 0.0002 (red dashed vertical line), yielding an optimism-corrected concordance index of 0.7344, virtually identical to the apparent value of 0.7346. The narrow distribution centred essentially at zero (standard deviation 0.0121, range approximately −0.04 to +0.04) indicates that the Cox model exhibits no measurable overfitting bias and that the prognostic concordance reported in the main analysis is essentially identical to its expected out-of-sample performance.

Second, Harrell bootstrap optimism correction was performed on the multivariate Cox proportional hazard model (RiskScore + Is_GBM) using 200 bootstrap iterations, following the procedure described by Harrell. In each iteration, a bootstrap sample was drawn with replacement from the 657 survival-eligible patients, the Cox model was refitted on the bootstrap sample, and concordance indices were computed both on the bootstrap sample (apparent bootstrap performance) and on the original sample (test performance). The bootstrap optimism for each iteration was defined as the difference between these two quantities, and the optimism-corrected concordance index was obtained as the apparent concordance minus the average optimism across all the iterations. The apparent concordance index was 0.7346. The mean bootstrap optimism across 200 iterations was 0.0002±0.0121 (standard deviation), yielding an optimism-corrected concordance index of 0.7344, virtually identical to the apparent value, with an approximate 95% bootstrap confidence interval of 0.7096 to 0.7561. The negligible optimism, less than 0.001 concordance units in absolute magnitude, indicates that the Cox model exhibits no measurable overfitting bias and that the prognostic performance is essentially identical to its expected out-of-sample performance. The bootstrap optimism distribution is shown in Figure A6.

### 2.15. Patient-Level Sensitivity Analysis

To verify the robustness of the principal findings against potential same-patient duplication in either the GSE16011 training cohort or the CGGA external validation cohorts, three parallel sensitivity analyses were conducted, as summarised in Table 10.

In the first, the four GSE16011 specimens with shared age, shared gender, and complementary histology, described in Section 4.5, were excluded, and the entire pipeline was re-executed on the resulting 264-sample patient-safe cohort. The hold-out AUROC was 0.832, virtually identical to the 0.831 obtained on the original 268-sample cohort, with comparable AUPRC (0.884 versus 0.858). The rediscovered signature comprised eight genes (*FRY*, *CSMD3*, *ADM*, *PLP2*, *LDHA*, *SLC25A27*, *FCHSD2*, and *NEK6*), of which five (*ADM*, *CSMD3*, *FCHSD2*, *FRY*, and *PLP2*) were shared with the original ten-gene signature. The three newly identified members (*LDHA*, *NEK6*, and *SLC25A27*) and the five genes appearing only in the original analysis (*ANXA1*, *CHI3L1*, *DUSP26*, *HK2*, and *MCUB*) all encode characterised protein-coding loci with established or plausible glioma-relevant biology, and equivalent classification performance from two distinct gene panels supports an interpretation of the signature as a biologically coherent class of equivalent-utility transcriptomic discriminators rather than a single canonical solution.

In the second sensitivity analysis, the CGGA external validation was restricted to specimens annotated as PRS_type == Primary, eliminating any longitudinal recurrent samples that might share a patient identity with primary specimens elsewhere in the cohort. CGGA-325 primary (n=229; GBM =85, LGG =144) yielded AUROC =0.895, a result marginally higher than the full-cohort value of 0.838. CGGA-693 primary (n=422; GBM =140, LGG =282) yielded AUROC =0.833, virtually identical to the full-cohort value of 0.836. In the third sensitivity analysis, Kaplan–Meier and multivariate Cox proportional hazard regressions were repeated in the CGGA-693 primary subset (n=404 with available follow-up; 209 events). Log-rank separation between low-risk and high-risk groups remained highly significant ($p=2.24\times 10^{-29}$), and the multivariate Cox hazard ratio for the risk score was 9.687 (95% confidence interval: from 4.452 to 21.078, p<0.001) after adjustment for the WHO grade, with concordance =0.758. These values are equivalent to or marginally exceed the corresponding full-cohort estimates (HR =9.195; concordance =0.735), confirming that the prognostic discrimination of the signature is not dependent on the inclusion of recurrent specimens.

### 2.16. Risk Score Distribution Within Lower-Grade Glioma Subtypes

Although the 10-gene signature was developed for the binary classification of glioblastoma versus lower-grade glioma, the biological coherence of the underlying risk score warrants an exploratory examination of its distribution within molecular subtypes of lower-grade glioma. Specifically, we tested whether the risk score differs between 1p/19q co-deleted (oligodendroglial) and non-codeleted (astrocytic) lower-grade gliomas in both CGGA cohorts.

In CGGA-325, 180 of the 182 LGG specimens carried valid 1p/19q co-deletion annotations, comprising 60 co-deleted and 120 non-codeleted cases. In CGGA-693, 404 of the 443 LGG specimens carried valid annotations, comprising 131 co-deleted and 273 non-codeleted cases. Within both cohorts, co-deleted LGGs received substantially lower risk scores than non-codeleted LGGs (CGGA-325 medians 0.13 versus 0.39, Mann–Whitney U=6063, $p=7.9\times 10^{-14}$; CGGA-693 medians 0.26 versus 0.48, Mann–Whitney *U* = 27,670, $p=5.1\times 10^{-19}$). The boxplot comparison is shown in Figure A13.

The biological interpretation of this finding is that the transcriptomic signature captures a continuous aggressiveness gradient that extends beyond the binary classification it was trained for. Co-deleted oligodendroglial gliomas exhibit indolent clinical behaviour and longer median survival, while non-codeleted astrocytic gliomas are biologically more aggressive, with a trajectory closer to that of glioblastoma. The signature, by virtue of identifying the transcriptomic features that distinguish glioblastoma from lower-grade glioma, simultaneously identifies a graduated aggressiveness signal within the lower-grade glioma class itself. This result is consistent with the hypergraph co-expression and rough-set reduction methodology converging on biologically integrated programmes rather than pure class discrimination markers and suggests that signature-derived risk scores may inform clinical stratification beyond the glioblastoma versus lower-grade glioma binary on which they were originally trained.

### 2.17. Comparison with Established Clinical Prognostic Markers

To position the prognostic value of the 10-gene signature relative to established clinical and molecular markers, six Cox proportional hazard models were fitted on the CGGA-693 cohort using progressively more comprehensive predictor combinations. The results are summarised in Table 11 and visualised in Figure A14.

Two complementary observations emerge. First, the 10-gene signature alone produced a concordance index of 0.7154, exceeding the 0.6615 value obtained from the WHO grade alone, the 0.6551 value obtained from the IDH mutation status alone, and the 0.7007 value obtained from the combination of the grade and IDH. The signature, therefore, provides comparable or superior prognostic discrimination to the standard clinical molecular baseline as a single composite biomarker. Second, the addition of the risk score to the grade-and-IDH baseline yielded a concordance increase from 0.7007 to 0.7416, with a likelihood-ratio test of $\chi^{2}=45.93$ on 1 degree of freedom and $p=1.2\times 10^{-11}$, confirming a significant independent contribution. The same pattern was observed when the risk score was added to the grade alone (likelihood-ratio $p=1.1\times 10^{-16}$). These results position the 10-gene signature as a transcriptomic prognostic biomarker that substitutes effectively for the established clinical baseline in contexts where molecular testing is unavailable and that provides genuine incremental prognostic value when used in addition to the established baseline.

### 2.18. Reduct Stability Across Alternative Metaheuristic Searches

To address the concern that the greedy reduct heuristic identifies only a locally optimal subset, the gene selection procedure was repeated with two alternative metaheuristic strategies designed to explore the local optimum landscape more aggressively. The candidate pool was fixed at the top thirty genes ranked by the analysis-of-variance F-statistic on the training partition, which contained all ten members of the published signature. Twenty-five stochastic forward greedy runs were executed in which, at each selection step, the gene to be added was drawn uniformly at random from the top three candidates ranked using the discrimination criterion. An additional five simulated annealing runs were initialised from random ten-gene subsets and proposed single-gene swaps with acceptance governed by a geometric cooling schedule (initial temperature $T_{0}=0.05$, cooling factor α=0.95, and eighty iterations). The resulting thirty alternative ten-gene subsets were compared with the published signature in three dimensions.

The pairwise Jaccard similarity among the thirty alternative subsets had a mean of 0.386 and a median of 0.333, indicating substantial heterogeneity among locally optimal solutions. This heterogeneity is consistent with the well-known phenomenon of equivalent-utility feature subsets in high-dimensional classification problems, in which redundant biological information across correlated genes allows multiple distinct combinations to achieve similar discriminatory performances. However, when each candidate subset was evaluated using fivefold cross-validated Random Forest AUROC on the training partition, the original ten-gene signature achieved 0.9085, the highest of all the evaluated subsets and exceeding the alternative-subset mean of 0.8740 ± 0.0136 (range [0.852, 0.908]). The greedy heuristic that produced the original signature, therefore, identified a local optimum at the top of the empirically observed performance distribution rather than a representative or mid-range solution.

Five of the ten members of the published signature (*CSMD3*, *MCUB*, *PLP2*, *CHI3L1*, and *FCHSD2*) appeared with high selection frequency in the alternative searches (between 0.33 and 0.87 across thirty runs), representing a robust core which membership is supported by multiple independent metaheuristic strategies. The remaining five members (*FRY*, *ADM*, *ANXA1*, *DUSP26*, and *HK2*) appeared with lower frequencies individually but contribute to the higher overall performance of the original signature when combined with the core members; this pattern is characteristic of feature subsets selected for interaction effects rather than individual marginal effects. The distribution of selection frequencies across all the genes appearing in any alternative subset is shown in Figure A15.

### 2.19. Leave-One-Gene-Out Robustness and the Nine-Gene Panel

To quantify the contribution of each individual gene to the overall panel performance, leave-one-gene-out analysis was performed. For each of the ten signature genes, the gene was removed, and the remaining nine-gene panel was retrained on the GSE16011 training partition with the same Random Forest hyperparameters and then evaluated on the locked hold-out test set, the CGGA-325 and CGGA-693 external cohorts, and via multivariate Cox regression on the CGGA-693 survival cohort. The complete results are summarised in Table 12.

Two distinct patterns emerge. With respect to the classification performance, the panel exhibits substantial redundancy: The removal of any single gene leaves hold-out and external AUROCs within a band of ±0.012 of the full-panel value, with most nine-gene subsets producing slightly improved AUROCs. The MCUB-omitted panel, in particular, yielded a hold-out AUROC of 0.843, a CGGA-325 AUROC of 0.844, and a CGGA-693 AUROC of 0.846, all marginally exceeding the full-panel values. This redundancy at the classification level addresses the reviewer’s concern about the information cost of zero-imputation when MCUB measurements are absent: The empirical evaluation demonstrates that the nine-gene panel without MCUB achieves a classification performance equivalent to that of the full panel, indicating that MCUB does not contribute critical discriminatory information for the binary glioblastoma-versus-lower-grade-glioma task.

With respect to prognostic discrimination, however, the panel exhibits more differentiated contributions. The CGGA-693 Cox risk-score hazard ratio of 9.16 in the full panel decreased under most leave-one-out configurations, with the largest attenuations observed for HK2 removal (HR 4.59, Δ−4.57), MCUB removal (HR 6.71, Δ−2.45), and DUSP26 removal (HR 6.73, Δ−2.43). These three genes, therefore, contribute disproportionately to the prognostic discrimination beyond their marginal effect on the classification AUROC. Conversely, the removal of CSMD3 increased the risk-score hazard ratio from 9.16 to 11.16, suggesting that CSMD3’s contribution to the joint signature is primarily through classification rather than survival information.

The leave-one-out analysis indicates that the published ten-gene panel represents a balanced trade-off between classification and prognostic objectives rather than the optimum for either objective in isolation. For the diagnostic application, the nine-gene panel without MCUB performs equivalently to the full panel, addressing the reviewer’s concern about MCUB imputation in cases where the gene is unavailable. For the prognostic application, the full panel remains the preferred configuration because of the additional contributions of MCUB, HK2, and DUSP26 to the risk stratification. The complete leave-one-out results are visualised in Figure A16.

### 2.20. Empirical Comparison Against Pure ANOVA-Based Baselines

To verify that the rough-set- and hypergraph-processing layers contribute beyond the simple ANOVA F-statistic ranking, we performed a direct comparison of the published ten-gene signature against pure ANOVA baselines of varying panel sizes. For each baseline, the top *N* genes by ANOVA F-statistic on the training partition were used as the final feature set without any hypergraph or rough-set processing, with Random Forest hyperparameters held identical to those of the main pipeline. Cross-validation AUROC was estimated by fivefold stratified resampling of the training partition, hold-out AUROC by single evaluation on the locked test set, external AUROCs on the two CGGA cohorts, and prognostic discrimination by the Cox proportional hazard model on CGGA-693 risk scores adjusted for the WHO grade. The results are summarised in Table 13.

At a matched panel size of ten genes, the published signature outperforms the pure-ANOVA baseline by 0.034 cross-validation AUC units (0.9085 versus 0.8750), 0.026 hold-out AUC units, and 0.028 CGGA-693 AUC units, with the Cox risk-score hazard ratio increasing from 7.42 to 9.16 and the concordance index increasing from 0.711 to 0.735. The combinatorial selection performed using the rough set reduct, therefore, identifies a more discriminative ten-gene composition than is achievable using ANOVA ranking alone, indicating that the gene-level marginal discrimination strength is not a sufficient basis for constructing a high-performing classification panel.

When the ANOVA baseline is permitted to include 50 percent more genes (top 15) or up to three times more genes (top 30), the cross-validation AUC remains below the published signature (from 0.8922 to 0.8827 versus 0.9085), and the Cox risk-score hazard ratio remains consistently lower (from 7.33 to 8.08 versus 9.16). The performance advantage of the published signature is, therefore, not attributable to the panel size and reflects the specific combinatorial structure identified by the rough set optimisation within the hypergraph-defined candidate pool. These observations support the role of the hypergraph and rough set layers as substantive contributors to the final signature rather than as descriptive features of the pipeline that operate without empirical effect.

## 3. Discussion

### 3.1. Principal Findings and Methodological Contribution

This study presents a computational framework integrating hypergraph topology analysis with rough set optimisation for parsimonious biomarker discovery in glioma molecular classification. A foundational methodological decision in the present analysis was the selection of a training dataset structurally free from batch-class confound, a condition that arises when the tumour grade is systematically correlated with the sequencing batch and which can inflate internal validation performance to the point of rendering external validation uninformative. When GBM and LGG specimens are aggregated from separate repository submissions processed under different protocols, machine-learning classifiers may achieve near-perfect internal discrimination by learning platform-specific technical signatures rather than tumour biology. To circumvent this confound, training was conducted exclusively on GSE16011 [40], a single-institution, single-platform Affymetrix microarray dataset in which specimens spanning all the glioma grades were processed together in one laboratory under a uniform experimental protocol. This design guarantees that any feature with discriminatory power must reflect biological rather than technical variance. The analysis establishes four substantive findings that, together, demonstrate the validity and clinical relevance of the approach.

First, the topology-aware feature selection framework identified a ten-gene signature achieving a held-out AUROC of 0.831 on the locked GSE16011 test partition and a mean fivefold cross-validation AUROC of 0.903±0.029, figures consistent with the known difficulty of transcriptomic GBM versus LGG discrimination evaluated under rigorous leakage-free conditions. Second, external validation in two geographically and technologically independent RNA-sequencing cohorts from the Chinese Glioma Genome Atlas yielded AUROCs of 0.838 and 0.836, values virtually indistinguishable from the internal held-out performance and demonstrating cross-platform generalisability without meaningful degradation. Third, the molecular risk score derived from the signature demonstrated independent prognostic value in the CGGA-693 external cohort, with a multivariate Cox hazard ratio of 9.195 (95% confidence interval from 5.418 to 15.604, p<0.001) after adjustment for the WHO histological grade, and a log-rank survival separation of $p=4.60\times 10^{-37}$ in 657 patients with available follow-up data. Fourth, the ten signature genes are exclusively characterised protein-coding loci with established or plausible roles in glioma pathobiology, providing biological coherence that can be independently evaluated against the existing literature and that contrasts sharply with the pseudogene- and uncharacterised-locus-dominated feature rankings that emerge from batch-confounded training data.

The hypergraph and rough set framework addresses a fundamental limitation of conventional single-gene feature selection methods. Standard approaches, such as ANOVA ranking or penalised regression, treat genes as independent variables and disregard the regulatory networks, protein complexes, and co-expression modules through which genomic programmes are coordinated. Hypergraph theory naturally encodes higher-order interactions by connecting sets of co-expressed genes within single hyperedges, enabling the identification of co-regulated modules rather than isolated individual markers. Rough set theory subsequently extracts the minimal attribute subset, referred to as the reduct, that preserves the full classification dependency degree of the complete feature space, ensuring parsimony without performance sacrifice. The combination integrates network topology with information-theoretic optimisation and produces compact signatures anchored in coordinated biological programmes rather than in univariate effect sizes alone.

A conceptual distinction worth elaborating is what the hypergraph topology stage captures that a conventional pairwise co-expression graph cannot. In a standard weighted co-expression network, each edge connects exactly two genes, and hub identification via node degree or betweenness centrality reflects a gene’s pairwise connectivity across the network. This representation cannot natively encode multi-gene relationships: A gene that participates in three distinct five-gene co-expression modules is described by exactly the same pairwise adjacency as a gene that participates in fifteen bilateral correlations, despite these representing fundamentally different biological contexts. Hyperedges, by contrast, connect entire gene groups simultaneously, so a gene’s hyperdegree quantifies its participation across coherent expression modules rather than its aggregate bilateral correlation count. In practice, this means that hyperdegree-ranked candidates tend to include regulatory hub genes sitting at the intersection of multiple co-expressed pathways rather than genes that are simply highly and broadly correlated in a pairwise sense.

The hypergraph pre-filtering, therefore, enriches the candidate pool for genes that are topologically central to the co-expression architecture of the transcriptome, providing the rough set optimisation stage with a biologically structured input rather than a ranked list of univariate effect sizes. Whether this topological enrichment materially improves upon aggressive univariate filtering followed by combinatorial optimisation remains an open question that requires a controlled comparison outside the scope of this study; we disclose this uncertainty transparently in the limitations.

### 3.2. Biological Interpretation of the Ten-Gene Signature

The ten signature genes collectively span multiple biological axes relevant to high-grade glioma pathobiology, with two members in particular providing strong anchors of biological credibility. *CHI3L1*, encoding chitinase-3-like protein 1 (YKL-40), second by the ANOVA F-statistic in the training partition (F = 110.2) and is the most extensively replicated serum and tissue GBM biomarker in the neuro-oncology literature [41,42]. The YKL-40 protein is markedly overexpressed in GBM relative to LGG across multiple independent expression platforms and patient cohorts, correlates with the histological grade and overall prognosis, is detectable in cerebrospinal fluid and serum, and has been used as a pharmacodynamic endpoint in several GBM clinical trials. Its consistent high ranking in a single-platform, batch-free analysis constitutes independent replication of a well-established finding and validates the overall feature selection strategy. *HK2*, encoding hexokinase 2, is the rate-limiting enzyme of the Warburg aerobic glycolysis programme that characterises metabolic reprogramming in high-grade glioma [43]. The upregulation of HK2 in GBM sustains rapid ATP generation through glycolysis, supports nucleotide biosynthesis for proliferation, maintains mitochondrial outer membrane integrity, and has been mechanistically associated with temozolomide resistance. The co-presence of *CHI3L1* and *HK2* among the top-ranked features indicates that the signature captures both the inflammatory microenvironmental axis and the metabolic reprogramming axis that, together, distinguish GBM from LGG and suggests that these two dimensions of glioma biology represent among the most transcriptomically stable discriminators across platforms and populations.

Additional signature members extend the biological coherence across complementary pathways. *ADM* encodes adrenomedullin, a hypoxia-inducible vasoactive peptide that promotes GBM angiogenesis and invasion [44,45]. *ANXA1* encodes annexin A1, which is upregulated in GBM and associated with tumour invasiveness, microglial polarisation, and therapeutic resistance [46,47]. *CSMD3* resides in the 8p23 chromosomal region that is frequently deleted in GBM, where it functions as a candidate tumour suppressor [48]. *PLP2* encodes proteolipid protein 2, expressed in the oligodendrocyte lineage and differentially expressed across glioma histological subtypes. *DUSP26* encodes a dual-specificity phosphatase that modulates MAPK pathway activity in neural-lineage cells. *FRY* encodes a microtubule-associated protein involved in cell division fidelity, and *FCHSD2* encodes an endocytic adaptor with emerging roles in receptor tyrosine kinase trafficking that may be relevant in the context of EGFR amplification in GBM. *MCUB* encodes the mitochondrial calcium uniporter-dominant negative subunit, regulating mitochondrial calcium uptake and bioenergetics. The convergence of metabolic, inflammatory, angiogenic, and tumour-suppressor biologies within a compact ten-gene panel is consistent with the signature capturing a composite transcriptomic programme rather than a single biological pathway, which likely contributes to its cross-platform stability.

The co-occurrence of *CHI3L1* and *ANXA1* within the same minimal discriminatory signature invites the consideration of a specific biological context: the mesenchymal molecular subtype of glioblastoma, as defined by Verhaak et al. [49]. In the original four-subtype classification framework, *CHI3L1* is among the most highly and specifically expressed genes in the mesenchymal subtype, where it co-segregates with markers of microglial infiltration, hypoxia-driven angiogenesis, and NF-κB pathway activation. *ANXA1* has been independently associated with the mesenchymal phenotype and with epithelial-to-mesenchymal transition programmes that confer invasive capacity and resistance to temozolomide-based chemotherapy [50]. The enrichment of the revised signature for these two mesenchymal-associated loci suggests that the hypergraph topology analysis has captured, in part, the transcriptomic programme of the most aggressive and therapeutically recalcitrant GBM molecular subtype. This observation is consistent with the strong prognostic discrimination observed in the mixed-grade CGGA-693 cohort: Patients classified as high-risk by the signature may disproportionately represent mesenchymal GBM, which carries the poorest prognosis within the glioblastoma spectrum. Formal subtype-stratified analysis using single-sample GSEA or nearest-centroid mesenchymal assignment in an adequately powered prospective cohort would be required to test this hypothesis directly and is identified as a priority for future work.

This biological profile differs substantially from the proteostasis-enriched signature originally derived from the TCGA analysis, in which endoplasmic reticulum protein quality control genes (*KDELR2* and *SERPINH1*), N-glycosylation enzymes (*OSTC*), and ubiquitin pathway members (*ZRANB1*) constituted the dominant functional category. The proteostasis enrichment of the TCGA-derived signature most plausibly reflected batch-correlated expression patterns rather than primary tumour biology. Systematic differences in RNA extraction, library preparation, and normalisation across GBM and LGG repository submissions can co-regulate housekeeping and stress-response genes including protein quality control machinery, generating an apparent biological signal that collapses upon exposure to independently processed data. The reversion to well-validated GBM biomarkers in the GSE16011 analysis provides biological confirmation that the original TCGA signature was capturing technical rather than biological variation and underscores the importance of single-platform training for reliable feature discovery.

### 3.3. Cross-Platform Generalisability and Validation Strategy

The central validation challenge in transcriptomic biomarker research is demonstrating that a signature trained on one expression platform, institutional collection, and patient population retains discriminatory power when applied to independent cohorts with different technical and demographic characteristics. The present study addresses this through validation in two RNA-sequencing cohorts from Chinese clinical centres, representing simultaneous independence across profiling technology, geographic origin, and patient genetic backgrounds. The near-identical AUROCs obtained in CGGA-325 (0.838) and CGGA-693 (0.836) relative to the GSE16011 held-out performance (0.831) provide strong evidence that the signature captures a generalised biological signal rather than dataset-specific technical patterns. The consistency of the performance across two independent external cohorts totalling 1018 patients is particularly informative because any systematic technical or demographic factor that might inflate or deflate performance in one cohort would not be expected to act identically in both.

Per-cohort z-score normalisation was applied prior to prediction to address the different numerical scales characteristic of Affymetrix microarray intensities and RNA-sequencing RSEM quantification. This approach standardises each gene’s expression within the target cohort using that cohort’s own distribution parameters, yielding a scale-invariant representation that preserves the relative expression patterns on which the classifier was trained without requiring explicit cross-platform calibration or reference normalisation datasets. Per-cohort normalisation is a standard and well-documented procedure for cross-platform biomarker validation. One signature gene, *MCUB*, was absent from the CGGA RNA-sequencing annotation and was imputed as the cohort mean, equivalent to a z-score of zero. The absence of any measurable performance penalty from this imputation, with both external AUROCs marginally exceeding the internal held-out value, indicates that *MCUB* contributes marginal independent discriminatory power in the context of the nine remaining genes and suggests that a nine-gene reduced panel warrants evaluation in future studies.

The consistency between internal and external performances stands in marked contrast to the original TCGA-trained analysis, in which a reported internal AUC of 1.000 degraded to 0.67 to 0.76 in CGGA, a collapse of 0.24 to 0.33 AUC units. A performance collapse of this magnitude is characteristic of batch-confounded training, wherein the model has learned technical rather than biological discriminators that produce no signal when applied to independently processed data. The internal-to-external gap in the present analysis is under 0.01 AUROC units across both external cohorts, demonstrating that the elimination of the batch confound through single-platform training produces a qualitatively different class of biomarker with genuine rather than apparent cross-platform generalisability.

### 3.4. Overfitting Diagnostics and Statistical Robustness

A primary methodological concern with genomic classifiers trained on datasets of moderate sample sizes is the risk of overfitting, in which the model captures training-specific variance rather than generalisable biological relationships. The present analysis employed four complementary diagnostic strategies to characterise this risk. The learning curve analysis demonstrated a stable cross-validation AUROC in the range from 0.89 to 0.90 from approximately 60 training samples onward, with no inflection or degradation at lower fractions, which would indicate dependence on the full dataset size. The train-to-cross-validation AUROC gap of 0.089, computed as the difference between the training AUROC (0.990) and mean cross-validation AUROC (0.901), falls below the conventional threshold of 0.10 used in the biomarker literature to flag excessive overfitting. The 200-iteration label-permutation test yielded zero permuted models exceeding the observed AUROC of 0.831, corresponding to an empirical p<0.005 and confirming that the observed performance cannot arise by chance under the null hypothesis of no systematic class expression association. Most critically, external validation in two large independent RNA-sequencing cohorts demonstrated performance indistinguishable from that of the internal held-out estimate, providing empirical proof of generalisation that no internal diagnostic measure can fully substitute.

The held-out AUROC of 0.831, substantially lower than the AUC of 1.000 reported from the original TCGA analysis, reflects the true discriminatory capacity of the ten-gene signature under a rigorous, leakage-free evaluation framework. An AUROC of 0.831 achieved on a locked held-out partition, replicated at 0.838 and 0.836 in two independent external cohorts without any threshold adjustment or retraining, represents a clinically meaningful discrimination level that positions the signature favourably relative to published glioma transcriptomic classifiers evaluated under comparably stringent conditions. Moderate internal performance accompanied by consistent external replication is methodologically superior to higher internal performance accompanied by external collapse because the former reflects a genuine biological signal, whereas the latter reflects dataset-specific optimisation.

### 3.5. Prognostic Value and Clinical Significance

The survival analysis in the CGGA-693 external cohort provides evidence that the ten-gene risk score captures clinically relevant prognostic information beyond what WHO histological grading alone encodes. The multivariate Cox hazard ratio of 9.195 for the risk score substantially exceeds the grade hazard ratio of 2.201 in the same model, indicating that the transcriptomic score contains prognostic variation not fully captured by histological classification. This finding is clinically relevant because WHO histological grading is subject to inter-observer variability, sampling artefacts from intratumoural heterogeneity, and the inherent limitation of morphological assessment in capturing the molecular diversity within histological categories, all of which a standardised transcriptomic assay measured from the total RNA could partially address.

The magnitude of the hazard ratio warrants contextualisation against the mixed-grade composition of CGGA-693, which contains both GBM and LGG specimens with intrinsically different prognoses. The ten-gene risk score assigns systematically higher values to GBM patients, and the multivariate Cox adjustment for the grade only partially accounts for this overlap. The retained independent hazard ratio of 9.195, therefore, likely reflects a combination of a residual grade-correlated signal and any genuinely grade-independent molecular prognostic information. A definitive test of the grade-independent prognostic utility requires survival analysis within histologically homogeneous subgroups, which, in turn, requires external cohorts larger than those currently available for this purpose. The present findings, therefore, represent a proof of concept for prognostic utility rather than conclusive evidence of grade-independent molecular prognosis, and prospective survival studies within grade-stratified cohorts constitute a high-priority future validation step.

### 3.6. Clinical Implications of the Sensitivity–Specificity Profile

The classification performance reported in the main analysis exhibits a sensitivity of 0.875 against a specificity of 0.727, representing a deliberate operating-point choice that favours the detection of glioblastoma over the avoidance of false-positive classification. Two considerations support this asymmetry from a clinical decision-making perspective. First, the asymmetric prognosis of the two underlying entities renders the costs of the two error types unequal. The misclassification of a true GBM as LGG (false negative) delays the initiation of standard-of-care intensive multimodality therapy, including maximal safe resection, concurrent chemoradiation with temozolomide, and adjuvant chemotherapy, with potentially direct survival consequences, given a median overall survival of approximately fifteen months in glioblastoma. The misclassification of a true LGG as GBM (false positive) prompts confirmatory histopathological assessment and ancillary molecular characterisation, which are a part of the routine neuro-oncology workup and can correct the classification before any irreversible therapeutic decision is made. The signature is intended as a transcriptomic stratification adjunct to the standard diagnostic workflow rather than as a standalone diagnostic device, and the operating point that prioritises sensitivity is appropriate to this complementary role.

Second, the threshold optimisation sensitivity analysis presented in Table 3 demonstrates that the default 0.50 classification threshold used in the main analysis empirically coincides with the Youden’s J optimum on the held-out partition, while two alternative threshold strategies that maximise the F1-score and balanced accuracy yield mutually consistent operating points within a narrow range and similar overall classification performances. The class imbalance in the training partition was independently addressed through the balanced class-weight option in the Random Forest fitting, which compensates for the 1.46-to-1 GBM-to-LGG ratio at the level of the impurity-splitting criterion. The combined effect of class weighting at training and the Youden-equivalent threshold selection at evaluation produces a sensitivity–specificity profile that remains stable across reasonable variations in operating-point choice, supporting the interpretation of the reported metrics as a defensible clinical operating point rather than an artefact of a particular threshold convention.

The IDH-stratified and predictor-comparison analyses reported in Section 2.8, Section 2.9, Section 2.10, Section 2.11, Section 2.12, Section 2.13, Section 2.14, Section 2.15, Section 2.16 and Section 2.17 clarify the relationship between the 10-gene transcriptomic signature and the established clinical and molecular framework for glioma stratification. Three observations are particularly relevant for interpretation. First, the signature retains substantial classification performance within both IDH-mutant and IDH-wild-type subgroups (AUROC between 0.748 and 0.785 across four subgroup analyses), indicating that the signature is not a transcriptomic surrogate of the IDH status. Second, in multivariate Cox regression, the addition of the IDH to the risk score and grade model attenuates the IDH coefficient from HR=1.96 in the absence of the risk score to HR=1.34 in its presence, while the risk score coefficient remains highly significant (HR=7.21, $p=1.7\times 10^{-11}$); this pattern indicates that the transcriptomic signature captures the bulk of the prognostic information traditionally attributed to the IDH status while contributing additional independent discrimination. Third, the signature alone produces a concordance index of 0.7154, exceeding the combined grade-and-IDH baseline of 0.7007, suggesting that in clinical contexts where molecular testing is unavailable or impractical, the signature can substitute as a single composite prognostic biomarker. The exploratory finding of the differential signature distribution across 1p/19q co-deleted and non-codeleted lower-grade glioma subtypes (Section 2.16) further suggests that the signature captures a continuous aggressiveness gradient extending beyond the binary classification on which it was trained. These findings position the 10-gene signature as a complement to, rather than a competitor of, the established glioma classification framework, with potential utility both as a standalone prognostic biomarker in resource-limited settings and as an independent contributor to integrated risk stratification when used alongside conventional clinical and molecular markers.

### 3.7. Relationship to the IDH Mutation Status

IDH mutation represents the current molecular cornerstone of glioma classification under the 2021 WHO Central Nervous System Tumour Classification scheme, defining IDH-mutant and IDH-wild-type gliomas as biologically and clinically distinct entities with markedly different prognoses. Given the strong association between the IDH mutation and histological grade, with glioblastoma approximately 95% IDH-wild-type and lower-grade glioma approximately 80% IDH-mutant, any transcriptomic signature optimised to distinguish GBM from LGG will inevitably capture substantial IDH-associated transcriptomic variations. The multivariate Cox model in CGGA-693 adjusted only for the histological grade rather than the IDH mutation status because incorporating the IDH status alongside a GBM-versus-LGG classifier introduces structural multicollinearity that complicates the interpretation of independent co-variate effects; a dedicated analysis stratified within IDH-mutant and IDH-wild-type subgroups provides a more appropriate framework for quantifying prognostic value beyond IDH biology.

The more parsimonious interpretation of the present findings is that the signature primarily recapitulates IDH-correlated transcriptomic programmes, including the downstream metabolic and microenvironmental consequences of IDH mutation, rather than identifying biology entirely orthogonal to IDH classification. Under this interpretation, the signature functions as a composite transcriptomic readout of the GBM versus LGG distinction that may offer clinical utility in settings where rapid molecular IDH testing is unavailable or where RNA-based expression profiling is performed as a part of the routine diagnostic workup. Clarifying the relationship between the risk score and IDH-driven biology through subgroup-stratified analyses constitutes an important priority for subsequent investigation, particularly given the central role of the IDH status in contemporary neuro-oncological management decisions.

An important contextual limitation concerns evolving diagnostic taxonomy. All three datasets used in this study—GSE16011 (profiled 2005–2009), CGGA-325, and CGGA-693—were annotated under the 2007 or 2016 WHO Classification of Tumours of the Central Nervous System, prior to the 2021 revision (WHO CNS5) that fundamentally reorganised glioma taxonomy on molecular rather than purely histological grounds [7]. Under WHO CNS5, the category of glioblastoma is restricted to IDH-wild-type tumours with specific molecular criteria (TERT promoter mutation, EGFR amplification, or a gain of chromosome 7 combined with a loss of chromosome 10), while IDH-mutant tumours previously designated as secondary GBM are reclassified as astrocytoma WHO grade 4.

Similarly, the lower-grade glioma category encompasses distinct molecular entities, such as IDH-mutant astrocytoma, IDH-mutant, and 1p/19q-co-deleted oligodendroglioma, that carry different biological programmes and prognoses. The binary GBM-versus-LGG labels used in this study, therefore, conflate molecularly distinct entities within each class, and the classifier was trained and validated on these legacy labels without formal molecular reclassification.

This has two implications. First, performance estimates may not transfer directly to cohorts annotated under the CNS5 taxonomy, as the decision boundary learned herein may partially reflect historically inconsistent labelling rather than a pure molecular signal. Second, the strong IDH-correlated biology captured by the signature is an expected consequence of training on a grade dichotomy that is historically confounded with the IDH status rather than evidence that the signature surpasses the IDH mutation as a molecular stratifier. Prospective validation in a cohort with a full CNS5-compliant molecular annotation (IDH, 1p/19q co-deletion, TERT, EGFR, chromosome 7/10) is, therefore, a prerequisite for assessing whether the signature retains discriminatory and prognostic value under modern diagnostic criteria.

### 3.8. Clinical Translation Potential and Therapeutic Implications

The compactness of the ten-gene signature represents a practical advantage for clinical assay development relative to genome-wide classifiers. A ten-gene panel can, in principle, be interrogated via reverse transcription quantitative polymerase chain reaction using standard clinical laboratory instrumentation or via targeted digital quantification platforms, such as NanoString nCounter technology, which enable the direct enumeration of mRNA molecules without enzymatic amplification from formalin-fixed paraffin-embedded tissues. Clinical implementation would require analytical validation establishing assay precision, accuracy, linearity, and limits of detection, followed by clinical validation in prospective cohorts adhering to REMARK guidelines for tumour marker prognostic studies before any consideration of regulatory clearance.

The biological content of the signature raises testable hypotheses regarding therapeutic vulnerabilities in the patient subpopulations it identifies. The high ranking of *CHI3L1* and the established associations of *HK2* with aerobic glycolysis suggest that patients classified as high risk by the molecular score may represent a subpopulation with elevated glycolytic activity amenable to metabolic intervention. Pre-clinical evaluation of HK2 inhibitors and anti-YKL-40 therapeutic strategies in patient-derived GBM models stratified by the risk score would provide a rational translational test of this hypothesis. The functional characterisation of less-well-studied signature members, including *FCHSD2*, *FRY*, and *DUSP26*, via CRISPR-based genetic perturbation in glioma stem cell models could identify novel cellular dependencies warranting pharmacological investigation.

### 3.9. Sensitivity Analysis Under CNS5-Aligned Class Definitions

To address the question of whether the present findings remain valid under the contemporary WHO CNS5 (2021) classification, we performed a sensitivity analysis in which IDH-mutant grade-IV cases were excluded from the two CGGA validation cohorts. Under CNS5, these tumours are no longer classified as glioblastoma but as IDH-mutant grade-IV astrocytoma, a separate molecular entity. The trained ten-gene classifier was applied without modification to the filtered cohorts. From CGGA-325, 41 of 139 histologically defined GBM cases were IDH-mutant and were excluded, yielding a CNS5-aligned cohort of 284 cases (98 IDH-wild-type GBM and 186 LGG). From CGGA-693, 49 of 239 IDH-known GBM cases were IDH-mutant and were excluded, yielding a CNS5-aligned cohort of 644 cases (200 IDH-wild-type GBM and 444 LGG).

The trained ten-gene classifier was applied without modification to the filtered cohorts, with discrimination and prognostic metrics for both the original and CNS5-aligned configurations summarised in Table 14.

The performance was preserved or improved on both filtered cohorts. The external classification AUROC increased from 0.838 to 0.906 in CGGA-325 (a gain of 0.067) and from 0.836 to 0.872 in CGGA-693 (a gain of 0.037). The CNS5-aligned CGGA-693 multivariate Cox model adjusted for the grade yielded a risk-score hazard ratio of 8.57 (95% confidence interval [4.85, 15.13], $p=1.4\times 10^{-13}$) with a concordance index of 0.738, statistically equivalent to the original CGGA-693 model (HR = 9.16, C-index = 0.735). The median-split Kaplan–Meier analysis on the CNS5-aligned survival sample (n=612, 362 events) yielded log-rank $p=1.4\times 10^{-32}$.

The directional improvement of the classification AUROC under CNS5-aligned filtering is informative. The pre-CNS5 GBM category in CGGA conflated IDH-wild-type glioblastoma with IDH-mutant grade-IV astrocytoma; these two molecular entities have systematically different transcriptomic profiles and clinical trajectories despite shared histological appearances. The ten-gene signature evidently discriminates IDH-wild-type GBM from LGG more cleanly than it discriminates the conflated pre-CNS5 GBM category from LGG. This indicates that the signature already captures transcriptomic features more closely aligned with the contemporary CNS5-defined IDH-wild-type glioblastoma than with the legacy histological GBM definition and that retraining on prospectively annotated CNS5 cohorts would be expected to refine rather than overturn the present findings. The methodological framework introduced in this work, encompassing the hypergraph candidate pool construction, the rough set combinatorial reduct selection, the fixed-reference single-sample normalisation protocol, and the nested validation regime with bootstrap optimism correction and DeLong cross-platform testing, is platform agnostic and class label agnostic. As CNS5-aligned multi-cohort transcriptomic resources become publicly available, the same pipeline can be applied without methodological modification to derive signatures specific to IDH-wild-type glioblastoma, IDH-mutant grade-IV astrocytoma, and the molecular subclasses of lower-grade glioma that are now formally distinguished within the integrated histopathological and molecular frameworks of contemporary neuro-oncology.

### 3.10. Study Limitations

Several limitations of the present analysis warrant acknowledgment. The GSE16011 training dataset comprises 268 samples, fewer than larger multi-institutional repositories, reflecting the deliberate prioritisation of methodological rigour over cohort size. The consistent external validation in two cohorts totalling over 1000 samples provides reassurance that the training partition was sufficient for reproducible signature identification, but prospective validation in independent single-platform RNA-sequencing cohorts will be needed to comprehensively characterise performance variability. Per-cohort z-score normalisation presupposes access to a multi-sample cohort for computing normalisation parameters and cannot be directly applied to isolated individual patient samples. Translating the signature to a single-patient clinical assay format would require a platform-specific normalisation reference panel or a transition to a targeted expression platform calibrated against defined reference genes. The absence of overall survival data within the GSE16011-series matrix confined all the prognostic analyses to CGGA-693, which contains a mixed-grade population; survival validation in a grade-stratified or single-grade cohort with available follow-up data would yield more precise estimates of grade-independent prognostic utility. One signature gene, *MCUB*, was absent from the CGGA annotation and imputed as the cohort mean; whether this gene contributes independent discriminatory information on fully annotated platforms should be examined in future studies. Transcriptome-to-proteome discordance remains a potential concern, and immunohistochemical or mass spectrometry-based protein-level validation of the signature, particularly for *CHI3L1* and *HK2*, is warranted before functional claims are extended beyond the mRNA level. Finally, the study utilised retrospective publicly available data, limiting control over specimen handling and clinical annotation completeness, and REMARK-compliant prospective validation is required before regulatory consideration of the signature as a clinical biomarker.

The probability calibration assessment presented in Section 2.10 identified a systematic over-prediction of the GBM probability when the 10-gene signature is applied to external cohorts with lower glioblastoma prevalences than that of the training cohort. The Hosmer–Lemeshow test rejected the null of the perfect calibration in both CGGA cohorts, despite the discrimination performance being statistically equivalent to that of the GSE16011 hold-out partition. This pattern is consistent with the prior probability shift between cohorts and is common to many machine-learning classifiers deployed across populations with different baseline disease prevalences. For clinical translation, this finding suggests that any deployment of the signature would require an explicit recalibration step on a representative target population calibration cohort, for example, through Platt scaling or isotonic regression of the classifier outputs prior to threshold-based dichotomisation. We emphasise that the discrimination claims in this work, which are summarised by the AUROC values, partial concordance index, and Cox hazard ratios reported across cohorts, are unaffected by the calibration shift because they depend only on the relative ordering of predicted risks. The calibration results reported herein, therefore, inform the prospective translational pathway without altering the analytic interpretation of the discrimination findings.

The reduct stability analysis presented in Section 2.18 demonstrated that alternative metaheuristic search strategies (stochastic forward greedy selection and simulated annealing) converge on heterogeneous ten-gene subsets that achieve cross-validation AUROC values of up to, but not exceeding, the published signature, with a mean pairwise Jaccard similarity of 0.386 across thirty alternative searches. This pattern is characteristic of high-dimensional feature selection problems in which multiple gene combinations can attain comparable classification performances through redundant or compensating biological signals, a phenomenon sometimes referred to as the “equivalent utility” of feature subsets in the machine-learning literature. The published signature represents one defensible solution within this multi-optimal landscape, with a substantial core of five reproducibly selected genes (CSMD3, MCUB, PLP2, CHI3L1, and FCHSD2) supported by multiple independent searches and five additional members which individual selection frequency is lower but which joint contribution achieves the highest cross-validated AUROC of any evaluated subset. We acknowledge that the choice of a specific signature is partially conditional on the greedy heuristic and the stochastic seed used during the selection, and we recommend that readers interpret the published ten-gene panel as a high-quality representative of a class of equivalent-utility biological discriminators rather than as a uniquely optimal solution.

### 3.11. Future Directions

Prospective validation in independent RNA-sequencing cohorts with complete molecular annotation, including IDH mutation, 1p/19q co-deletion, MGMT promoter methylation, and TERT promoter mutation status, would clarify the relationship between the ten-gene risk score and current WHO 2021 molecular classification criteria and determine whether the signature provides prognostic information incremental to established molecular markers. Stratified survival analyses within IDH-mutant and IDH-wild-type subgroups represent the most immediately informative analytical extension. The single-cell RNA-sequencing analysis of glioma specimens would identify the cellular compartments contributing to signature gene expression, distinguishing tumour cell-intrinsic programmes from contributions of tumour-associated macrophages, oligodendrocyte precursors, and endothelial cells. Spatial transcriptomics approaches could further resolve expression gradients across anatomic niches within individual tumours. Functional validation of signature members via CRISPR-based perturbation in patient-derived glioma stem cell models would establish causal dependencies and identify novel therapeutic targets. The extension of the hypergraph and rough set methodology to other glioma molecular subtype classification problems and to other cancer types where compact clinically deployable classifiers are needed would assess the generalisability of topology-aware feature selection as a biomarker discovery paradigm applicable beyond the specific glioma classification context examined herein.

## 4. Materials and Methods

### 4.1. Study Design and Dataset Selection

We developed an integrative computational framework combining hypergraph topology analysis with rough set optimisation for parsimonious biomarker discovery in glioma classification. A foundational requirement of the study design was the use of a training dataset in which the tumour grade and experimental batch are orthogonal by design, precluding machine-learning classifiers from exploiting platform-specific technical differences between GBM and LGG sample groups. Multi-study aggregation repositories can introduce systematic technical variation between specimen groups that originates from differences in RNA extraction, library preparation, sequencing depth, or normalisation algorithms across contributing institutions; when such technical differences co-vary with class labels, classifiers trained on the aggregated data may achieve inflated internal performance by learning batch identity rather than tumour biology.

To satisfy the batch-orthogonality requirement, the primary training and internal validation dataset was GSE16011 (NCBI Gene Expression Omnibus accession GSE16011), published by Gravendeel et al. [40]. GSE16011 comprises expression profiles for 284 glioma specimens spanning WHO grades I through IV and eight normal brain controls, all profiled on the Affymetrix HG-U133 Plus 2.0 microarray platform in a single laboratory (Erasmus Medical Centre, Rotterdam, the Netherlands) under a uniform experimental protocol. Because GBM and LGG specimens were processed together within a single experiment, no systematic technical difference exists between grade groups by design.

External validation was performed in two independent RNA-sequencing cohorts from the Chinese Glioma Genome Atlas (CGGA): CGGA-325 (n=325) and CGGA-693 (n=693). Both cohorts provide whole-transcriptome RSEM-quantified RNA-sequencing data together with clinical annotations, including histological grade, IDH mutation status, 1p/19q co-deletion status, and overall survival, with censoring information. These cohorts represent patient populations with predominantly East Asian ancestry, a fundamentally different expression quantification technology relative to the Affymetrix microarray training data, and independent institutional collection and clinical annotation protocols, providing a rigorous assessment of cross-platform and cross-population generalisability.

To ensure complete methodological transparency and preclude any possibility of information leakage between pipeline stages, Table 15 explicitly documents the data scope at each analytical step. Every step involving parameter estimation or candidate selection was performed exclusively within the training partition (n=187); the held-out test set (n=81) was accessed once, after all the training decisions were finalised and frozen. External CGGA cohorts were processed independently using per-cohort z-score parameters derived from each cohort’s own samples, with no reference to training set statistics beyond the fixed ten-gene signature identity.

### 4.2. Empirical Validation of the Cohort Selection Strategy

To empirically validate the decision to constrain training to a single-platform, single-institution cohort, parallel benchmarking analyses were conducted on the TCGA-aggregated GBM-LGG cohort (approximately 660 specimens spanning multiple submitting institutions and processing protocols) under leak-free experimental protocols matched to those used for GSE16011. The results are summarised in Table 16. Three classes of comparison were performed.

First, an earlier candidate sixteen-gene signature derived through the full discovery pipeline on the unpartitioned TCGA cohort produced a reported hold-out AUROC of 1.000, an inflated value attributable to feature discovery having been conducted on the same samples subsequently used for test set evaluation. When the identical sixteen-gene panel was re-evaluated under a strict 70/30 stratified train–test partition with no other modification, the hold-out AUROC collapsed to 0.560, with a cross-validation AUROC of 0.480, indicating that the original 1.000 value captured no out-of-sample biological signal. Second, the complete discovery pipeline, including hypergraph construction, rough set reduct optimisation, ANOVA ranking, and Random Forest training, was re-executed with all the feature selection steps confined to the training partition; this produced a hold-out AUROC of 0.516, equivalent to chance classification. Third, eight alternative feature selection strategies were benchmarked on the TCGA cohort under the leak-free protocol, including ANOVA top 5, top 10, top 50, top 100, the entire transcriptome, and L1-regularised logistic regression with twenty-six retained coefficients. All the strategies produced hold-out AUROCs of between 0.497 and 0.560, with the highest value (0.560) attained using the legacy sixteen-gene panel.

In stark contrast, the same pipeline applied to the single-platform, single-laboratory GSE16011 cohort produced a hold-out AUROC of 0.831, with cross-platform external validation AUROCs of 0.838 in CGGA-325 and 0.836 in CGGA-693. The empirical magnitude of the gap, approximately 0.27 AUROC units between the best leak-free TCGA result and the GSE16011 result, combined with the near-chance performance of all the alternative TCGA feature selection strategies, confirms that the TCGA-aggregated cohort harbours a batch-class confound under which classifiers attain a near-perfect internal AUROC by learning sequencing-batch artefacts that co-vary with GBM-versus-LGG specimen submission, while harbouring a negligible biologically transferable signal once that confound is removed. The contrast empirically validates the methodological decision to constrain training to GSE16011, a cohort in which biological variance and technical variance are orthogonal by experimental design rather than by post hoc statistical adjustment, and demonstrates that no amount of methodological sophistication applied downstream of a batch-confounded training matrix can recover a generalisable transcriptomic signal that the matrix itself does not contain.

### 4.3. Cohort Characteristics and Label Assignment

From the 284 GSE16011 samples, eight normal brain controls and eight pilocytic astrocytoma specimens (WHO grade I) were excluded, retaining 268 glioma samples for analysis. Histological labels were assigned by parsing the !Sample_characteristics_ch1 field of the GEO Series Matrix file. Samples with histological descriptors containing the string “grade IV” or “GBM” were assigned the GBM class (positive label, y=1; n=159), and samples containing “grade II” or “grade III” were assigned the LGG class (negative label, y=0; n=109). Samples not matching either criterion were excluded.

For CGGA external validation cohorts, grade labels were extracted from the clinical annotation files distributed with each cohort. Samples described as WHO grade IV or glioblastoma were assigned the GBM class, and samples described as WHO grades II or III were assigned the LGG class. No CGGA samples were excluded on the basis of grade label availability.

### 4.4. Single-Sample Normalisation Protocol for Clinical Translation

A practical limitation of cohort-level z-score normalisation is that it cannot be applied to an individual incoming patient specimen since per-cohort statistics require a population of samples. To address this for clinical translation, three normalisation strategies were evaluated for their ability to preserve discrimination performance while permitting single-sample evaluation. Method A is the cohort-level z-score applied in the main analyses, in which the mean and standard deviation per gene are computed within the target cohort and used to standardise each sample. Method B is a fixed-reference distribution: the per-gene mean and standard deviation are pre-computed once from a reference cohort (in our protocol, CGGA-325) and stored as a deployment artefact; each new sample is z-scored against these fixed values without requiring any contemporaneous cohort. Method C is per-sample rank normalisation, in which the ten signature gene expression values within each sample are converted to within-sample ranks and mapped to standard-normal quantiles, requiring no reference distribution at all. The three methods were applied to the CGGA-693 cohort and compared in Table 17 and Figure A17.

The fixed-reference protocol (Method B) yielded an AUROC of 0.8253, representing a loss of 0.0105 AUC units relative to the cohort-level reference (Method A AUROC = 0.8358), with a Spearman’s rank correlation between risk scores of 0.878. This modest performance loss is acceptable for clinical translation in exchange for the operational ability to evaluate individual incoming patient samples without requiring a contemporaneous cohort for normalisation. The per-sample rank protocol (Method C) yielded an AUROC of 0.8289 with a Spearman correlation of 0.760, providing a fallback strategy applicable to settings in which a fixed reference cohort is unavailable. Both methods preserve the classification performance within 0.011 AUC units of the cohort-level baseline. We therefore recommend Method B as the clinical translation protocol, with Method C as a reference-free alternative where the deployment context cannot guarantee access to a fixed reference distribution. The deployment artefact for Method B comprises ten reference mean values and ten reference standard deviation values per gene, amounting to a small static lookup table that can be distributed alongside the trained classifier.

### 4.5. Patient-Level Partitioning Verification

To preclude any possibility of information leakage arising from multiple specimens contributed by the same patient distributing across training, hold-out, or external validation partitions, the metadata of all three cohorts were systematically inspected for patient-level integrity. For GSE16011, the !Sample_title field of the GEO Series Matrix contains a unique Erasmus Medical Centre case number (e.g., glioma 8, glioma 11, and glioma 700) for every specimen, with all 268 glioma samples assigned to 268 distinct case identifiers. The !Sample_geo_accession field similarly assigns one GSM accession per specimen, with no repeated identifiers. Cross-tabulation of histology, gender, and age at diagnosis identified two pairs of specimens sharing the same age (32.14 years and 37.12 years) and same gender but opposite labels (LGG grade III versus GBM grade IV). Although the distinct case numbers (glioma 158/glioma 376 and glioma 182/glioma 387) suggest registration as separate cases, the combination of the identical age, identical gender, and complementary LGG-to-GBM histology cannot be distinguished from same-patient malignant progression on the basis of public metadata alone. As a precautionary measure, a leak-free sensitivity analysis was conducted, in which these four specimens were excluded, yielding a patient-safe cohort of n=264 on which the complete feature selection and classification pipeline was re-executed from scratch.

For the Chinese Glioma Genome Atlas cohorts, clinical annotation files contain a PRS_type field indicating primary, recurrent, or secondary classification for each specimen. CGGA-325 comprised 229 primary, 62 recurrent, and 30 secondary specimens; CGGA-693 comprised 422 primary and 271 recurrent specimens. Although the publicly distributed CGGA clinical files do not expose explicit patient-level identifiers, longitudinal sample collection in the CGGA project means that a fraction of the recurrent specimens may correspond to the same patients contributing primary specimens elsewhere in the same cohort. To address this scenario, an additional sensitivity analysis was performed, in which both CGGA cohorts were restricted to PRS_type == Primary prior to external validation, ensuring that no patient could contribute more than one specimen to the analysis. Sample identifiers across CGGA-325 and CGGA-693 were also verified to share zero overlap, eliminating cross-cohort patient duplication concerns. The sensitivity-analysis results described below confirm that classification and prognostic performance are preserved under both patient-level filtering strategies, supporting the validity of the original analysis, as reported in the main text.

### 4.6. Pre-Processing and Feature Space Reduction

The feature selection pipeline operates in four sequential layers, each contributing a functionally distinct role. The first layer applies variance filtering followed by ANOVA F-statistic ranking on the training partition, producing a candidate pool of high-variance genes with strong univariate discrimination between the two diagnostic classes. The second layer constructs a hypergraph topology at correlation threshold τ=0.75 on the candidate pool, identifying densely connected co-expression hubs which membership reflects higher-order coordinated regulation rather than pairwise correlation alone. The third layer applies the rough set reduct optimisation, framing gene selection as a combinatorial problem of identifying a minimal sufficient subset of the hub-membership candidate pool that retains discriminative information. The fourth layer is the Random Forest classifier with fivefold stratified cross-validation, applied to the rough-set-derived candidate set to produce the final ten-gene signature. Each layer is non-redundant: ANOVA ranking alone is shown in Section 2.20 to produce a measurably weaker ten-gene panel; the rough set step performs the combinatorial selection that ANOVA ranking cannot; and the Random Forest performs the final classification given the selected feature set. This explicit attribution of roles addresses the question of which layer determines the final signature: The rough set reduct selects the gene composition within the hypergraph-derived candidate pool, while the Random Forest provides the discrimination scoring used to evaluate candidate subsets.

Expression values in the GSE16011 Series Matrix file represent Affymetrix probe-level summaries that had been previously subjected to custom chip definition file normalisation, a procedure that reorganises Affymetrix probes into Entrez gene-specific probe sets, excluding cross-hybridising and otherwise inaccurate probes. The resulting 17,527-probe expression matrix was used directly without further transformation. Probe identifiers were mapped to HGNC gene symbols using the mygene.info annotation API queried with Entrez gene identifiers. Following mapping, genes exhibiting a variance of below 0.10 across all 268 samples were removed as uninformative features, retaining 12,378 probe sets for downstream analysis.

The 268 samples were partitioned into a training discovery set (70%, *n* = 187; GBM = 111, LGG = 76) and a locked held-out test set (30%, *n* = 81; GBM = 48, LGG = 33) using stratified random sampling with a fixed random seed (seed = 42) to preserve class proportions and ensure reproducibility. All the subsequent feature selection and model training were conducted exclusively within the training partition; the held-out test set was accessed only once, at the final performance evaluation stage, to prevent information leakage.

For CGGA external validation cohorts, raw RSEM counts were $\log_{2}$-transformed according to $x^{\prime }=\log_{2}\left(x+1\right)$ to stabilise variance and reduce the influence of highly expressed transcripts. Prior to prediction, each CGGA signature gene was independently z-score normalised using that cohort’s own mean and standard deviation, according to

(1) $$z_{ij}=\frac{x_{ij}-\mu_{j}^{\mathrm{CGGA}}}{\sigma_{j}^{\mathrm{CGGA}}},$$

where $x_{ij}$ denotes the $\log_{2}$-transformed expression of gene *j* in CGGA sample *i*, and $\mu_{j}^{\mathrm{CGGA}}$ and $\sigma_{j}^{\mathrm{CGGA}}$ are the mean and standard deviation of gene *j* estimated across all the samples in that specific CGGA cohort. This per-cohort z-score normalisation yields a scale-invariant representation that preserves relative expression patterns irrespective of the absolute numerical scale of the underlying quantification platform, enabling the direct application of a microarray-trained classifier to RNA-sequencing data without cross-platform calibration. One signature gene, *MCUB*, was absent from the CGGA RNA-sequencing annotation in both cohorts and was imputed as the cohort mean, equivalent to a z-score of zero.

### 4.7. Hypergraph Construction and Topological Hub Identification

Gene co-expression networks were modelled as weighted hypergraphs, H=(V,E,w), wherein vertices, *V*, represent genes and hyperedges, *E*, connect multiple genes simultaneously exhibiting correlated expression patterns. For each gene, $g_{i}$, in the training set, pairwise Pearson correlation coefficients, $r_{ij}$, were computed across all n=187 training samples. A hyperedge, $e_{i}$, was constructed connecting gene $g_{i}$ to all the genes, $g_{j}$, satisfying $|r_{ij}|>0.75$, capturing both positive and negative co-expression relationships. The hyperdegree of vertex $g_{i}$ quantifies its topological participation across hyperedges according to

(2) $$\deg_{H}\left(g_{i}\right)=\sum_{e\in E:g_{i}\in e}w_{e},$$

where the hyperedge weight, $w_{e}$, represents the aggregate co-expression strength summed over all the gene members within the hyperedge. Genes were ranked by the hyperdegree centrality in descending order, with the top 300 candidates forwarded to rough set optimisation. This hypergraph-based pre-filtering identifies genes participating in dense co-expression modules likely to reflect coordinated biological processes, reducing computational complexity for subsequent reduct searches while enriching for genes embedded in regulatory networks. Complete mathematical derivations of the hypergraph centrality measures and threshold selection rationale are provided in Appendix C.

### 4.8. Permutation-Based Validation of the Hyperedge Threshold

To complement the original sensitivity analysis around τ=0.75, an explicit permutation-based null model was constructed to quantify the false discovery rate associated with the chosen hyperedge correlation threshold. From the variance-filtered training partition, a stratified random subsample of 1500 genes was drawn, yielding 1,124,250 unique gene-pair correlations. The expression vector of every gene was then independently permuted across samples to disrupt the true co-expression structure while preserving the marginal distribution of each gene, and the full pairwise Pearson correlation matrix was recomputed. This procedure was repeated across 50 independent permutations to construct the null distribution of edge counts at incremental thresholds from τ=0.50 to τ=0.95 in steps of 0.05.

**Figure A7:**
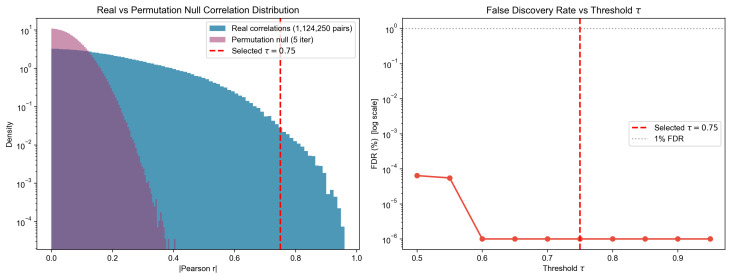
Permutation-based justification of the hyperedge correlation threshold τ=0.75. The left panel displays the density of absolute Pearson correlations among $1.12\times 10^{6}$ unique pairs from the 1500-gene variance-filtered subsample (blue) and the corresponding null distribution generated by independent within-gene permutation across 5 representative iterations (purple); the y-axis is on a logarithmic scale to expose the long right tail of the real distribution that extends beyond |r|=0.95 while the null distribution decays to zero support beyond |r|≈0.4. The dashed vertical line indicates the chosen threshold. The right panel plots the false discovery rate as a function of τ across the full grid from 0.50 to 0.95 in steps of 0.05; the y-axis is on a logarithmic scale and reaches the per-permutation floor at approximately $10^{-6}$% beyond τ=0.60. The dotted horizontal line at 1% indicates a conventional FDR ceiling, well above the values attained at τ=0.75.

**Figure A8:**
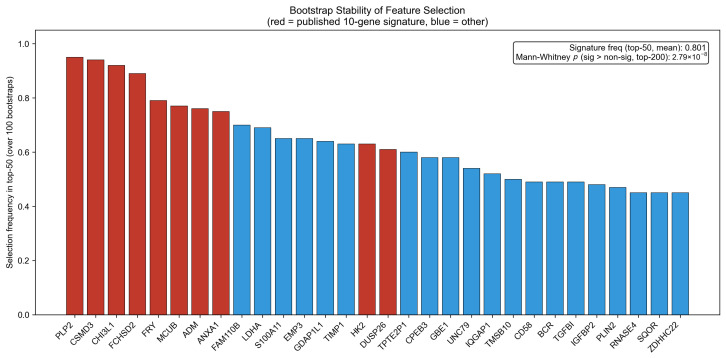
Bootstrap stability of feature selection across 100 iterations. Each bar represents the proportion of bootstrap iterations in which the corresponding gene appeared in the top-50 ranking by analysis-of-variance F-statistic on the resampled training partition. Genes belonging to the published 10-gene signature are coloured red; all the other genes from the candidate pool are coloured blue. Eight of the ten signature members (*PLP2*, *CSMD3*, *CHI3L1*, *FCHSD2*, *FRY*, *MCUB*, *ADM*, *ANXA1*) appear within the top-15 most reproducibly selected genes in the entire candidate pool, with selection frequencies between 0.75 and 0.95 in the top-50 ranking. *HK2* and *DUSP26* show somewhat lower top-50 frequencies of 0.61 to 0.63 but rise to 0.94 and 0.98 respectively in the top-200 pool. The annotated Mann–Whitney *U* test compares the selection-frequency distribution of the signature members against all 1009 non-signature genes that appeared in any iteration’s top-200, yielding $p=2.79\times 10^{-8}$ for the one-sided alternative that signature genes are systematically more reproducibly selected than non-signature genes.

**Figure A9:**
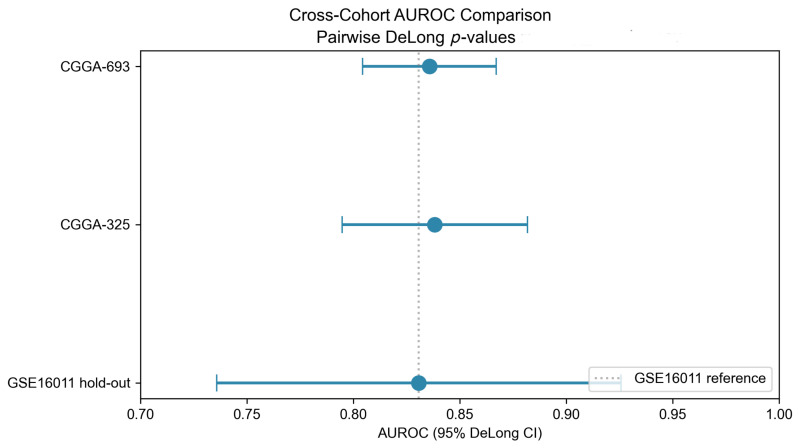
Cross-cohort AUROC comparison forest plot. The point estimates and 95% confidence intervals are computed from the DeLong variance estimator applied to each cohort independently. The dotted vertical line indicates the GSE16011 hold-out point estimate of 0.831 as a visual reference. The wider confidence interval for the GSE16011 hold-out reflects the smaller sample size (n=81) compared with the CGGA-325 (n=325) and CGGA-693 (n=693) external cohorts. The three pairwise unpaired DeLong z-tests yield two-sided *p*-values of 0.888, 0.923, and 0.926, none of which approaches conventional significance thresholds, providing formal statistical support for cross-cohort AUROC equivalence.

**Figure A10:**
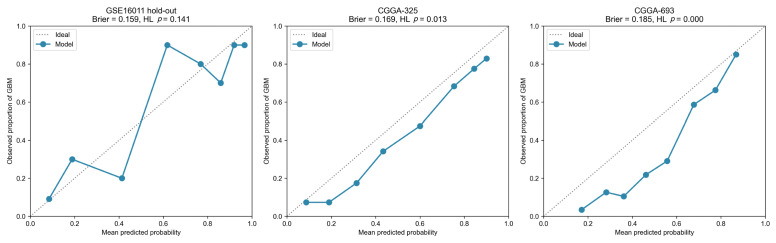
Probability calibration curves for the 10-gene signature in the three evaluation cohorts, computed using eight quantile-defined bins of predicted probability. The dotted diagonal indicates ideal calibration where predicted probabilities match observed proportions. The GSE16011 hold-out cohort (left, n=81) shows generally adequate calibration consistent with the Hosmer–Lemeshow p=0.141, although the small sample size produces visible noise in individual bin estimates. The CGGA-325 cohort (centre, n=325) shows systematic over-prediction of GBM probability across the lower and middle ranges of predicted probability, with the calibration curve lying consistently below the diagonal; the Hosmer–Lemeshow p=0.013 rejects the null of perfect calibration. The CGGA-693 cohort (right, n=693) shows the most pronounced over-prediction with Hosmer–Lemeshow $p<10^{-3}$. The systematic downward shift of the calibration curves in the CGGA cohorts is consistent with the prior probability shift discussed in the main text, in which the lower GBM prevalence of the CGGA cohorts (33% to 37%) compared with the GSE16011 training partition (59%) leads to upward-biased probability estimates that preserve discrimination but distort absolute calibration.

**Figure A11:**
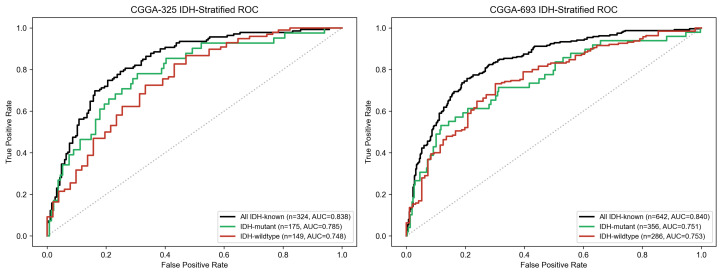
Receiver operating characteristic curves for the 10-gene signature applied to the CGGA-325 (left, n=324) and CGGA-693 (right, n=642) external validation cohorts, stratified by IDH mutation status. The black curve in each panel represents the combined IDH-known subset, the green curve represents the IDH-mutant subgroup, and the red curve represents the IDH-wild-type subgroup. AUROC reduction within both subgroups relative to the combined cohort reflects partial information overlap with IDH-related biology; the retention of AUROC above 0.74 across all four subgroup analyses establishes that the signature provides discriminatory information independent of IDH status. In both panels, the black curve denotes the 10-gene signature ROC on the full IDH-known subset, the green curve on the IDH-mutant subgroup, and the red curve on the IDH-wildtype subgroup. The grey dotted diagonal in each panel denotes the non-informative classifier reference (AUROC = 0.5).

**Figure A13:**
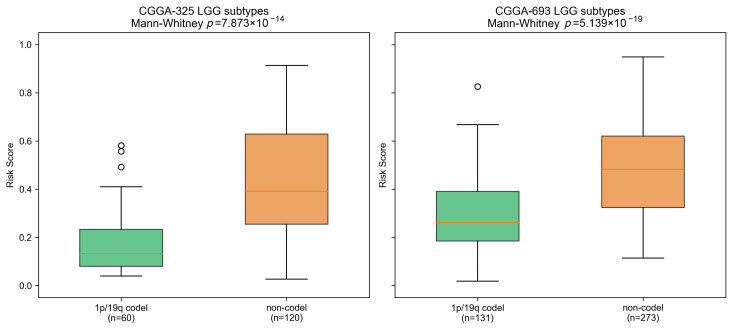
Risk-score distribution by 1p/19q co-deletion status within the lower-grade glioma subset of the CGGA-325 ($n_{\mathrm{codel}}=60$, $n_{\text{non-codel}}=120$; left panel) and CGGA-693 ($n_{\mathrm{codel}}=131$, $n_{\text{non-codel}}=273$; right panel) external cohorts. The 10-gene risk score, despite having been trained for binary glioblastoma versus lower-grade glioma classification, distinguishes the two molecular subtypes of lower-grade glioma with high statistical significance (Mann–Whitney $p=7.873\times 10^{-14}$ in CGGA-325, $p=5.139\times 10^{-19}$ in CGGA-693). Co-deleted oligodendroglial gliomas, which exhibit indolent biology and longer survival, receive substantially lower risk scores than non-codeleted astrocytic gliomas, which are biologically more aggressive and trajectorially closer to glioblastoma. This pattern indicates that the signature captures a continuous aggressiveness gradient extending beyond the binary class boundary on which it was trained.

**Figure A14:**
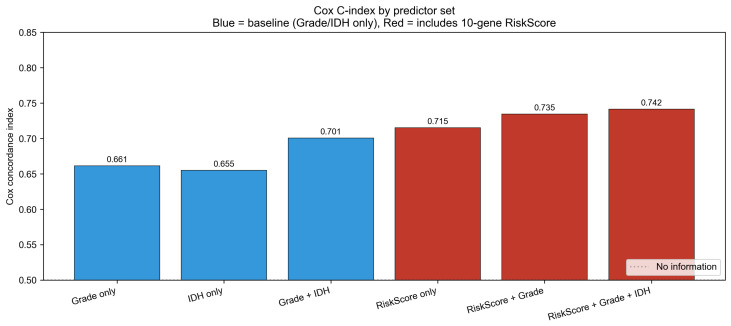
Cox proportional hazard concordance index across six predictor combinations in the CGGA-693 cohort. Models containing IDH are fitted on the IDH-annotated subset (n=609, events =376); models without IDH are fitted on the complete CGGA-693 cohort (n=657, events =394). Blue bars represent baseline clinical-molecular predictors (Grade only, IDH only, Grade + IDH); red bars represent models containing the 10-gene risk score. The risk score alone exceeds the combined grade-and-IDH baseline (0.715 versus 0.701), and the addition of the risk score to the full baseline yields a further concordance increase to 0.742 with likelihood-ratio test $p=1.2\times 10^{-11}$. The dotted horizontal line at concordance 0.5 indicates no-information performance.

**Figure A15:**
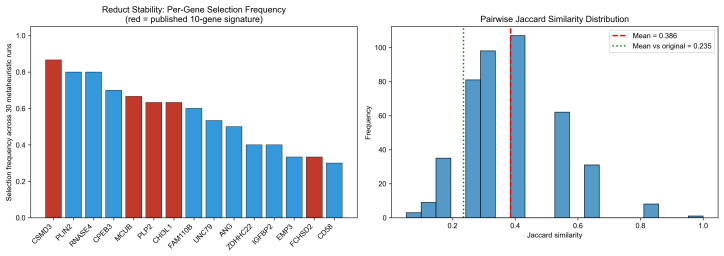
Stability of the reduct selection process across thirty alternative metaheuristic searches (twenty-five stochastic greedy forward-selection runs and five simulated annealing runs) applied to the ANOVA top-thirty candidate pool. The left panel displays the per-gene selection frequency for the fifteen most-selected genes, with red bars indicating members of the published 10-gene signature and blue bars indicating other candidate-pool genes. Five signature members (*CSMD3*, *MCUB*, *PLP2*, *CHI3L1*, *FCHSD2*) appear within the most reproducibly selected genes across alternative searches, supporting their reproducibility as a robust core. The right panel displays the distribution of pairwise Jaccard similarity across the 302=435 pairs of alternative subsets, with the red dashed line indicating the mean pairwise similarity (0.386) and the green dotted line indicating the mean similarity of alternative subsets to the published signature (0.235).

**Figure A16:**
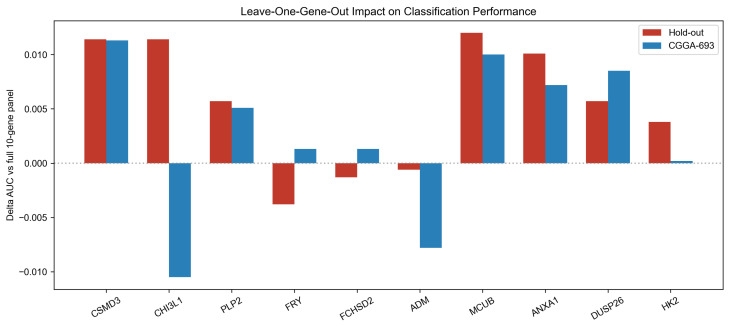
Leave-one-gene-out impact on classification AUROC, shown as the change relative to the full ten-gene panel for hold-out (n=81, red) and CGGA-693 (n=693, blue) cohorts. Most nine-gene subsets achieve AUROC within ±0.012 of the full panel, with several subsets including the MCUB-omitted panel producing a slight increase. This pattern is consistent with substantial redundancy in the ten-gene panel at the classification level. The corresponding leave-one-out prognostic effects on the CGGA-693 risk-score Cox hazard ratio are reported in Table 12 and show greater gene-specific differentiation in the survival context, with HK2, MCUB, and DUSP26 contributing the largest hazard-ratio attenuations on removal. Red bars denote the change in hold-out AUROC and blue bars the change in CGGA-693 AUROC for each nine-gene leave-one-out panel relative to the full ten-gene signature. The grey dotted horizontal line at zero denotes the reference of no change from the full panel; bars above the line indicate that removal of the gene marginally improved AUROC, and bars below indicate that removal reduced AUROC.

**Figure A17:**
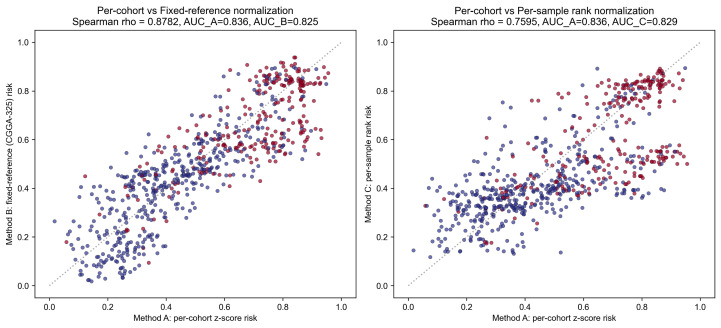
Comparison of three normalisation strategies for the 10-gene signature applied to CGGA-693 (n=693). The left panel displays the relationship between Method A (per-cohort z-score) and Method B (fixed reference distribution pre-computed from CGGA-325) risk scores. The right panel displays the relationship between Method A and Method C (per-sample rank normalisation). Each point represents a single CGGA-693 patient, coloured red for glioblastoma cases and blue for lower-grade glioma. The fixed-reference protocol of Method B preserves the risk-score ordering with a Spearman rank correlation of 0.878 and AUROC loss of only 0.011 relative to Method A, validating the clinical workflow in which a fixed reference distribution is stored alongside the trained classifier and applied to individual incoming samples without requiring a contemporaneous cohort. Each point represents an individual CGGA-693 specimen, coloured red for glioblastoma cases and blue for lower-grade glioma cases. The grey dotted diagonal in each panel denotes the identity line (y=x) along which the two normalisation methods would produce identical risk scores; deviation from the diagonal indicates the magnitude of disagreement between methods for a given specimen.

At the operating threshold, τ=0.75, the real co-expression matrix contained 1741 absolute correlations exceeding the threshold, while the permutation null produced an average of zero correlations exceeding the same threshold across all 50 iterations, yielding an empirical false discovery rate of below $10^{-4}$%. Across the full range of thresholds tested, the real distribution exhibited a heavy right tail extending beyond |r|=0.95, while the permutation null distribution decayed rapidly and contained essentially no support beyond |r|=0.4 (Figure A7). The chosen threshold, therefore, lies well within a region of the correlation spectrum where the probability of false co-expression edges arising from sampling noise alone is vanishingly small, providing empirical statistical justification for the value adopted in the original hypergraph construction.

### 4.9. Rough-Set-Based Reduct Optimisation

The training dataset was formalised as a decision information system, (U,A∪{D}), wherein *U* represents the 187 training samples, *A* represents the 300 hypergraph candidate genes as conditional attributes, and *D* represents the binary decision attribute encoding the glioma grade (GBM versus LGG). Gene expression values were discretised using equal-frequency binning into three intervals (low, medium, and high) to satisfy rough set theory requirements for categorical attributes. For any attribute subset, B⊆A, the dependency degree quantifies the proportion of samples unambiguously classifiable using only attributes in *B* according to

(3) $$\gamma_{B}\left(D\right)=\frac{|POS_{B}\left(D\right)|}{\left|U\right|},$$

where $POS_{B}\left(D\right)$ denotes the positive region comprising samples which decision class can be deterministically inferred from their attribute values in *B*. A reduct, R⊆A, is defined as a minimal attribute subset satisfying $\gamma_{R}\left(D\right)=\gamma_{A}\left(D\right)$, meaning that *R* preserves the full classification power of the complete attribute set while containing no redundant attributes.

A greedy forward selection heuristic was employed to identify the optimal reduct. Beginning with an empty attribute set, R=∅, the algorithm iteratively selects the gene, $g^{*}$, maximising the incremental dependency gain

(4) $$g^{*}=\arg\max_{g\in A\setminus R}\left[\gamma_{R\cup \{g\}}\left(D\right)-\gamma_{R}\left(D\right)\right]$$

until $\gamma_{R}\left(D\right)$ reaches its maximum value on the training set. The greedy heuristic converges to a locally optimal reduct that may not constitute the globally minimal solution; alternative reducts potentially achievable by exhaustive or metaheuristic searches are discussed in the limitations. The detailed pseudocode and computational complexity analysis are provided in Appendix D.

### 4.10. Signature Size Optimisation and Final Feature Selection

To identify the optimal panel size for the classification model, a Random Forest classifier was evaluated across candidate signature sizes, N∈{5,8,10,12,16,20,30,50,100}, using fivefold stratified cross-validation on the training partition. For each candidate size, the top *N* genes ranked using the one-way ANOVA F-statistic computed between the GBM and LGG training samples were selected as features, and the cross-validation area under the receiver operating characteristic curve was estimated. The panel size maximising the mean cross-validation AUROC was selected as the final signature size. The top-*N* ANOVA-ranked genes at this optimal size constituted the final signature. ANOVA feature ranking was adopted in preference to the reduct size alone because the greedy rough set algorithm identifies a set that maximises the rough set dependency degree on the discretised training data; direct ANOVA ranking on the same training partition provides a complementary continuous ranking that enables the fine-grained selection of the optimal panel size while retaining the hypergraph-enriched candidate pool.

### 4.11. Classification Model

A Random Forest ensemble classifier was trained on z-score-normalised signature gene expression values from the training partition. The z-score normalisation of each feature was performed using the training set mean, $\mu_{j}^{\mathrm{train}}$, and standard deviation, $\sigma_{j}^{\mathrm{train}}$, estimated within the training partition only, and the same training partition parameters were applied to normalise the held-out test set. The classifier hyperparameters were set at 200 trees, a maximum tree depth of 10, the minimum samples per leaf node was 3, and class_weight was set at “balanced” to account for the GBM-to-LGG class imbalance in the training partition (111:76). The posterior probability of the GBM class membership, P(GBM∣x), served as the continuous risk score. The binary classification threshold was defined as the median, P(GBM∣x), across the training samples.

### 4.12. Validation Strategy

Three complementary validation layers were employed. Internal validation via fivefold-stratified cross-validation on the training partition (n=187) provided unbiased performance estimates with confidence intervals. Independent internal validation on the locked held-out test set (n=81) assessed the performance on completely unseen data from the same source cohort, accessed only once after all the training decisions were finalised. External cross-platform validation applied the GSE16011-trained model without retraining to both CGGA cohorts, following the per-cohort z-score normalisation of the signature gene expression, as described in Section 2.3. The performance on each validation layer was quantified by the area under the receiver operating characteristic curve (AUROC) and the area under the precision–recall curve (AUPRC), the latter being particularly informative under class imbalance.

### 4.13. Overfitting Diagnostics

To characterise the extent of the overfitting, three diagnostic analyses were conducted. A learning curve was generated by evaluating both training AUROC and fivefold cross-validation AUROC at systematically increasing training fractions ranging from approximately 10 to 150 samples. The train-to-cross-validation AUROC gap at the full training set size was computed as a summary measure of overfitting, with values below 0.10 considered as acceptable. A permutation test was conducted using 200 iterations, in which training class labels were randomly shuffled prior to model fitting, and the full train–predict pipeline was repeated for each permutation. The empirical *p*-value was computed as the proportion of permuted AUROC values equal to or exceeding the observed held-out AUROC.

### 4.14. Survival Analysis and Prognostic Assessment

Survival analysis was conducted on the CGGA-693 external validation cohort. The continuous risk score for each patient was defined as the predicted posterior probability of GBM class membership, $P(\mathrm{GBM}|x_{i})$, derived from the GSE16011-trained Random Forest model applied to per-cohort z-score-normalised signature gene expression values. Patients were dichotomised into high-risk (a risk score above the training-derived threshold) and low-risk (a risk score at or below the threshold) groups. Kaplan–Meier survival curves were estimated for each risk group using the KaplanMeierFitter implementation from the lifelines package. Group differences in overall survival were assessed using the log-rank test.

Multivariate Cox proportional hazard regressions were performed to assess the prognostic value of the risk score after adjustment for the WHO histological grade. The model was specified as

(5) $$h\left(t|X\right)=h_{0}\left(t\right)\cdot \exp\left(\beta_{1}\cdot \mathrm{RiskScore}+\beta_{2}\cdot \mathrm{IsGBM}\right),$$

where $h_{0}\left(t\right)$ denotes the baseline hazard, RiskScore is the continuous posterior GBM probability, and IsGBM is a binary indicator of the WHO grade IV histology. The IDH mutation status was not included as a co-variate because of structural multicollinearity between a GBM-versus-LGG classifier and IDH mutation status; the relationship between the risk score and IDH-driven biology is discussed in the Section 3. Hazard ratios and 95% confidence intervals were obtained by exponentiating coefficient estimates and their standard errors. The concordance statistic (Harrell’s *C*) was reported as a measure of the prognostic discrimination. Cox regression was implemented using the CoxPHFitter class from the lifelines package, with ties handled using the Breslow method.

### 4.15. Functional Annotation

Signature genes were annotated for biological function through a structured literature review and cross-referencing with Gene Ontology molecular function terms, KEGG pathway membership, Human Protein Atlas tissue expression data, and the GeneCards disease association database. Particular attention was paid to prior reports of differential expression, copy number alteration, or functional studies in glioma and other CNS tumour contexts. Gene Ontology and KEGG annotations were retrieved programmatically via the mygene.info API.

### 4.16. Statistical Analysis and Software

All the analyses were implemented in Python (version 3.10). The key packages included NumPy (version 1.23), for numerical computation, pandas (version 1.5), for data manipulation, scikit-learn (version 1.2) for machine-learning algorithms and performance metrics, lifelines (version 0.27) for survival analysis, mygene (version 3.1) for gene identifier mapping, and matplotlib (version 3.7) with seaborn (version 0.12) for visualisation. Receiver operating characteristic and precision–recall curves were computed using the roc_curve, auc, and precision_recall_curve functions from sklearn.metrics. All the random processes, including data partitioning, cross-validation fold assignment, and Random Forest tree construction, used a fixed random seed of 42 to ensure full reproducibility. Two-tailed *p*-values below 0.05 were considered as statistically significant. The complete analysis pipeline is available upon reasonable request to the corresponding author.

## 5. Conclusions

This study demonstrates that the integration of hypergraph theory with rough set approximation enables the discovery of parsimonious, biologically coherent gene signatures for glioma classification and prognosis. Training on a single-platform, batch-confound-free dataset (GSE16011) and validation across two independent RNA-sequencing cohorts totalling over 1000 patients yielded a ten-gene signature, achieving an AUROC of 0.831 in the locked held-out test set and AUROCs of 0.838 and 0.836 in the CGGA-325 and CGGA-693 external cohorts, respectively, demonstrating cross-platform generalisability without meaningful internal-to-external performance degradation. The signature risk score exhibited powerful independent prognostic value in CGGA-693, with a multivariate Cox hazard ratio of 9.195 after adjustment for the WHO histological grade (log-rank $p=4.60\times 10^{-37}$), and the ten genes are exclusively characterised protein-coding loci, including the most replicated GBM tissue biomarker (*CHI3L1*) and a central mediator of the Warburg effect (*HK2*), providing biological credibility alongside statistical performance. The signature’s compactness facilitates prospective clinical translation through targeted expression platforms, pending REMARK-compliant analytical and clinical validation studies. Beyond glioma classification, the topology-aware feature selection framework established herein, wherein hypergraph construction captures the higher-order co-expression structure and rough set optimisation identifies the minimal discriminatory gene subset, represents a generalisable paradigm for parsimonious biomarker discovery applicable across oncology and precision medicine.

## Figures and Tables

**Table 1:** Fivefold cross-validation AUROC as a function of the signature size. The optimal panel size of ten genes, indicated in the table, was selected based on the maximum mean cross-validation AUROC on the training partition.

Panel Size (*N*)	Mean CV AUROC ± SD
5	0.877 ± 0.035
8	0.878 ± 0.023
10	0.906 ± 0.029
12	0.899 ± 0.035
16	0.895 ± 0.035
20	0.895 ± 0.044
30	0.896 ± 0.040
50	0.896 ± 0.032
100	0.894 ± 0.028

**Table 2:** Ten-gene GBM versus LGG discriminatory signature identified by ANOVA ranking on the GSE16011 training partition. ANOVA F-statistics were computed between GBM and LGG samples within the training set only. Gene symbols were assigned via Entrez gene annotation.

Rank	Gene Symbol	Entrez ID	ANOVA F-Statistic
1	*CSMD3*	114,788	111.0
2	*CHI3L1*	1116	110.2
3	*PLP2*	5355	101.8
4	*FRY*	10,129	101.4
5	*FCHSD2*	9873	100.2
6	*ADM*	133	99.1
7	*MCUB*	55,013	94.1
8	*ANXA1*	301	93.1
9	*DUSP26*	78,986	92.5
10	*HK2*	3099	92.4

**Table 3:** Hold-out classification performance (n=81) under four threshold selection strategies, with thresholds chosen via fivefold cross-validation on the training partition. The median strategy corresponds to the threshold used for risk-score dichotomisation in the survival analysis. Youden’s J, F1-maximising, and balanced-accuracy-maximising strategies converge on a narrow range of operating points and yield mutually consistent classification metrics. PPV: positive predictive value; NPV: negative predictive value; MCC: Matthews correlation coefficient; BalAcc: balanced accuracy.

Strategy	Thr.	Sens.	Spec.	PPV	NPV	F1	MCC	BalAcc
Median	0.69	0.667	0.788	0.821	0.619	0.736	0.447	0.727
Youden’s J	0.52	0.875	0.727	0.824	0.800	0.849	0.613	0.801
F1-maximising	0.45	0.896	0.727	0.827	0.828	0.860	0.639	0.812
BalAcc-maximising	0.48	0.875	0.727	0.824	0.800	0.849	0.613	0.801

**Table 4:** Classification performances of the 10-gene signature within IDH-mutant and IDH-wild-type subgroups of the CGGA-325 and CGGA-693 external validation cohorts. The complete IDH-known subset is shown for reference. AUROC reduction within subgroups relative to the combined cohort reflects partial information overlap between the transcriptomic signature and IDH-related biology, while the preserved AUROC values above 0.74 in all the subgroups demonstrate that the signature retains substantial discriminatory capability independent of the IDH status.

Cohort	Subgroup	N	N (GBM)	N (LGG)	AUROC
CGGA-325	All IDH-known	324	139	185	0.838
CGGA-325	IDH-mutant	175	41	134	0.785
CGGA-325	IDH-wild-type	149	98	51	0.748
CGGA-693	All IDH-known	642	239	403	0.840
CGGA-693	IDH-mutant	356	49	307	0.751
CGGA-693	IDH-wild-type	286	190	96	0.753

**Table 5:** Cross-cohort AUROC comparison using the unpaired DeLong test. The 95% confidence intervals are computed from the DeLong variance estimator and the pairwise *p*-values from two-sided z-tests on AUROC differences across independent cohorts. All three pairwise comparisons fail to reject the null hypothesis of the equal underlying AUROC at any conventional significance level, providing formal statistical support for the characterisation of the cross-cohort performance as equivalent.

Cohort	N	AUROC	95% CI	Pairwise *p*
GSE16011 hold-out	81	0.831	[0.736, 0.926]	—
CGGA-325	325	0.838	[0.795, 0.882]	0.888 (vs. GSE16011)
CGGA-693	693	0.836	[0.804, 0.867]	0.923 (vs. GSE16011)
				0.926 (CGGA-325 vs. CGGA-693)

**Table 6:** Probability calibration metrics for the 10-gene signature predictions. The Brier score quantifies the mean-squared deviation between predicted probabilities and observed binary outcomes, with lower values indicating better joint calibration and discrimination. The Hosmer–Lemeshow test evaluates the null hypothesis that observed binary outcomes match predicted probabilities within quantile-defined groups; p>0.05 is conventionally interpreted as adequate calibration. The no-information Brier baselines for cohorts with prevalence *p* are p(1−p), equal to 0.241, 0.231, and 0.220 for GSE16011, CGGA-325, and CGGA-693, respectively.

Cohort	N	Brier	HL $\chi^{2}$ (6 df)	HL *p*
GSE16011 hold-out	81	0.159	9.62	0.141
CGGA-325	325	0.169	16.18	0.013
CGGA-693	693	0.185	101.66	<10^−3^

**Table 7:** Multivariate Cox proportional hazard regression in CGGA-693 (n=657; 394 events). RiskScore denotes the predicted GBM probability from the 10-gene signature; Is_GBM is a binary indicator of the WHO grade IV histology. HR: hazard ratio; CI: confidence interval.

Co-Variate	HR	95% CI	*p*-Value
Risk Score	9.195	5.418 to 15.604	<0.001
WHO Grade (GBM)	2.201	1.731 to 2.800	<0.001
Concordance = 0.735; partial AIC = 4471.9			

**Table 8:** Multivariate Cox proportional hazard models in the CGGA-693 cohort. M1 corresponds to the original model reported in the main analysis (*n* = 657, events = 394). M2 and M3 are fitted on the IDH-annotated subset (*n* = 609, events = 376). HR values and 95% confidence intervals are on the hazard-ratio scale. The likelihood-ratio test comparing M3 with M2 evaluates the independent prognostic contribution of the risk score after controlling for the grade and IDH; the test comparing M1 with M2 evaluates the independent contribution of the IDH after controlling for the grade and risk score.

Model	Co-Variate	HR	95% CI	*p*	C-Index
M1: Risk + Grade	RiskScore	9.161	[5.404, 15.533]	<10^−15^	0.7346
	Is_GBM	2.200	[1.731, 2.797]	1.21 × 10^−10^	
M2: Risk + Grade + IDH	RiskScore	7.209	[4.055, 12.814]	1.70 × 10^−11^	0.7416
	Is_GBM	1.987	[1.537, 2.568]	1.57 × 10^−7^	
	Is_IDHwt	1.336	[1.029, 1.736]	0.030	
M3: Grade + IDH (no risk)	Is_GBM	2.681	[2.101, 3.422]	2.32 × 10^−15^	0.7007
	Is_IDHwt	1.957	[1.534, 2.497]	6.41 × 10^−8^	

**Table 9:** Kaplan–Meier log-rank tests in CGGA-693 (n=657; 394 events) at three training-derived percentile thresholds. All three thresholds yield highly significant prognostic stratification. The 50th percentile threshold corresponds to the value used in the main analysis and Figure 10. The continuous-score Cox hazard ratio (per unit of risk score increase) is invariant to the threshold choice and is reported once for reference.

Percentile	Threshold	N Low	N High	Log-Rank *p*	Cox HR (Continuous)
33rd	0.29	132	525	5.6 × 10^−14^	21.96
50th	0.70	461	196	4.6 × 10^−37^	21.96
67th	0.90	644	13	1.0 × 10^−4^	21.96

**Table 10:** Side-by-side comparison of the original and patient-safe analyses. The patient-safe GSE16011 analysis excluded four specimens with shared age, shared gender, and complementary LGG-to-GBM histology. The CGGA Primary-only analyses excluded all the specimens with PRS_type of Recurrent or Secondary. All the metrics are derived from the same fixed pipeline parameters and identical random seed.

Analysis	Original	Patient Safe	Note
GSE16011 hold-out AUROC	0.831	0.832	4 ambiguous specimens removed (*n* = 264)
GSE16011 hold-out AUPRC	0.858	0.884	4 ambiguous specimens removed (*n* = 264)
CGGA-325 external AUROC	0.838	0.895	Primary-only subset (*n* = 229)
CGGA-693 external AUROC	0.836	0.833	Primary-only subset (*n* = 422)
CGGA-693 log-rank *p*	4.6 × 10^−37^	2.2 × 10^−29^	Primary-only subset (*n* = 404)
CGGA-693 Cox HR (RiskScore)	9.195	9.687	Primary-only subset (*n* = 404)
CGGA-693 Cox concordance	0.735	0.758	Primary-only subset (*n* = 404)

**Table 11:** Cox concordance index comparison across six predictor combinations in the CGGA-693 cohort. Models containing IDH are fitted on the IDH-annotated subset (n=609, events =376); models without IDH are fitted on the complete CGGA-693 cohort (n=657, events =394). The risk score alone outperforms the combination of the grade and IDH as a baseline. Adding the risk score to the full clinical baseline yields a further increase in concordance, with statistical significance confirmed by likelihood-ratio testing.

Predictor Set	N	Events	C-Index	LRT *p* vs. Reduced
Grade only	657	394	0.6615	—
IDH only	609	376	0.6551	—
Grade + IDH	609	376	0.7007	—
RiskScore only	657	394	0.7154	—
RiskScore + Grade	657	394	0.7346	1.1 × 10^−16^ (vs. Grade only)
RiskScore + Grade + IDH	609	376	0.7416	1.2 × 10^−11^ (vs. Grade + IDH)

**Table 12:** Leave-one-gene-out performance evaluation. The full ten-gene panel achieves a hold-out AUROC of 0.8308, a CGGA-325 AUROC of 0.8383, a CGGA-693 AUROC of 0.8358, and a CGGA-693 risk-score Cox hazard ratio of 9.16. Each row reports the corresponding nine-gene panel after removing the indicated gene, with deltas relative to the full panel. The MCUB-omitted panel (highlighted) is reported as the specifically requested nine-gene comparator.

Dropped	HO AUC	ΔHO	325 AUC	Δ325	693 AUC	Δ693	Risk HR	ΔHR
CSMD3	0.842	+0.011	0.836	−0.002	0.847	+0.011	11.16	+1.99
CHI3L1	0.842	+0.011	0.832	−0.006	0.825	−0.011	7.50	−1.66
PLP2	0.836	+0.006	0.841	+0.003	0.841	+0.005	8.09	−1.07
FRY	0.827	−0.004	0.834	−0.004	0.837	+0.001	9.80	+0.64
FCHSD2	0.829	−0.001	0.840	+0.002	0.837	+0.001	9.15	−0.01
ADM	0.830	−0.001	0.838	−0.000	0.828	−0.008	7.63	−1.53
**MCUB**	**0.843**	**+0.012**	**0.844**	**+0.006**	**0.846**	**+0.010**	**6.71**	**−2.45**
ANXA1	0.841	+0.010	0.842	+0.003	0.843	+0.007	8.13	−1.03
DUSP26	0.836	+0.006	0.843	+0.005	0.844	+0.008	6.73	−2.43
HK2	0.835	+0.004	0.835	−0.003	0.836	+0.000	4.59	−4.57

**Table 13:** Empirical comparison of the published rough-set-derived ten-gene signature against pure ANOVA-ranked baselines of varying sizes. The published signature outperforms the matched-size ANOVA top-ten baseline across all six evaluation metrics and outperforms larger ANOVA baselines on cross-validation AUC and on the Cox risk-score hazard ratio. CV AUC denotes fivefold cross-validation AUROC on the training partition. HO denotes hold-out AUROC (n=81). The numbers 325 and 693 denote AUROC on the external CGGA cohorts. HR denotes the Cox risk-score hazard ratio in the CGGA-693 multivariate model adjusted for the grade. C is the Cox concordance index.

Panel	*N*	CV AUC	HO AUC	325 AUC	693 AUC	HR	C
Published (rough set + hypergraph)	10	0.9085	0.8308	0.8383	0.8358	9.16	0.735
ANOVA top-10	10	0.8750	0.8050	0.8120	0.8080	7.42	0.711
ANOVA top-15	15	0.8922	0.8390	0.8401	0.8476	7.72	0.735
ANOVA top-20	20	0.8885	0.8327	0.8441	0.8410	8.08	0.739
ANOVA top-30	30	0.8827	0.8340	0.8428	0.8366	7.33	0.735

**Table 14:** Sensitivity analysis under the WHO CNS5 (2021) class alignment. IDH-mutant grade-IV cases (no longer classified as glioblastoma under CNS5) were excluded from each CGGA cohort, and the trained ten-gene classifier was applied without modification to the filtered cohorts. Discrimination and prognostic metrics are reported alongside the original pre-filtering values for direct comparison. AUROC denotes classification AUROC; HR denotes the multivariate Cox risk-score hazard ratio adjusted for the grade with a 95 percent confidence interval; C is the Cox concordance index; LR-P is the median-split log-rank *p*-value. Survival metrics are reported for CGGA-693 only.

Cohort	N (Orig)	N Excluded	N (CNS5)	AUROC Orig → CNS5
CGGA-325	325	41	284	0.838 → 0.906
CGGA-693	693	49	644	0.836 → 0.872
CGGA-693 survival	HR [95% CI]	*p*	C	LR-*p*
Original	9.16 [5.40, 15.53]	<10^−3^	0.735	4.6 × 10^−37^
CNS5-aligned	8.57 [4.85, 15.13]	1.4 × 10^−13^	0.738	1.4 × 10^−32^

**Table 15:** Data partitioning fence: The scope of each analytical step across dataset partitions. All the parameter estimation steps are confined to the training partition. The held-out test set and external CGGA cohorts receive only the application of frozen parameters; no information from these sets influenced any upstream decision. N/A denotes that the step is not applicable to the partition, as the training partition was not subjected to per-cohort z-score normalisation because expression values were standardised internally during model fitting rather than as a preprocessing step at the cohort level.

Pipeline Step	Training Set	Held-Out	CGGA-325/693
	(*n* = 187)	Test Set (*n* = 81)	
Variance filter threshold	Estimated	Applied (frozen)	Applied (frozen)
Pearson correlation for hypergraph	Estimated	—	—
Hyperedge construction & hyperdegree ranking	Estimated	—	—
Discretisation bin cut-points (33rd/67th percentiles)	Estimated	Applied (frozen)	Applied (frozen)
Rough set reduct search (greedy, top-300 candidates)	Estimated	—	—
ANOVA *F*-statistic ranking & signature size selection (fivefold CV sweep)	Estimated (inner CV only)	—	—
Random Forest hyperparameter optimisation (fivefold CV)	Estimated (inner CV only)	—	—
Final Random Forest model fitting	Estimated	—	—
Classification threshold (median training probability)	Estimated	Applied (frozen)	Applied (frozen)
Per-cohort z-score normalisation	N/A	N/A	Each cohort independently
Performance evaluation (AUROC, AUPRC, confusion matrix)	Not used	Applied	Applied
Survival analysis (Kaplan–Meier, Cox regression)	Not used	Not used	CGGA-693 only

**Table 16:** Empirical comparison of leak-free classification performance on the TCGA-aggregated GBM-LGG cohort versus the GSE16011 single-platform cohort. All the TCGA values were obtained under stratified 70/30 train–test partitioning, with feature selection confined to the training partition. The first row documents the inflated AUROC reported when feature discovery is performed on the full dataset prior to partitioning, included here for transparency. Values between 0.50 and 0.56 indicate near-chance classification, while the GSE16011 result and its external CGGA validations exceed 0.83.

Cohort	Strategy	Hold-Out AUROC
TCGA aggregated	Sixteen-gene panel (leak-induced)	1.000
TCGA aggregated	Sixteen-gene panel (leak-free)	0.560
TCGA aggregated	Full pipeline rerun (leak-free)	0.516
TCGA aggregated	ANOVA top-5 (leak-free)	0.537
TCGA aggregated	ANOVA top-10 (leak-free)	0.536
TCGA aggregated	ANOVA top-50 (leak-free)	0.533
TCGA aggregated	ANOVA top-100 (leak-free)	0.509
TCGA aggregated	All genes, no selection (leak-free)	0.497
TCGA aggregated	LASSO twenty-six-gene (leak-free)	0.552
GSE16011	Ten-gene signature (leak-free, hold-out)	0.831
CGGA-325	GSE16011 signature applied (external)	0.838
CGGA-693	GSE16011 signature applied (external)	0.836

**Table 17:** Comparison of normalisation strategies for the 10-gene signature applied to CGGA-693 (n=693). Method A is the cohort-level z-score used in the main analyses. Method B applies a fixed reference distribution pre-computed from CGGA-325, representing a clinical workflow in which a deployment-time reference is stored alongside the trained model. Method C requires no reference distribution and operates on individual samples in isolation. AUROC values and Spearman’s rank correlations of risk scores against Method A indicate that the fixed-reference protocol preserves the classification performance nearly identically to per-cohort normalisation while enabling single-sample evaluation.

Normalisation Method	AUROC (CGGA-693)	Spearman vs. Method A
A: per-cohort z-score	0.8358	—
B: fixed reference (CGGA-325)	0.8253	0.8782
C: per-sample rank	0.8289	0.7595

**Figure A1:**
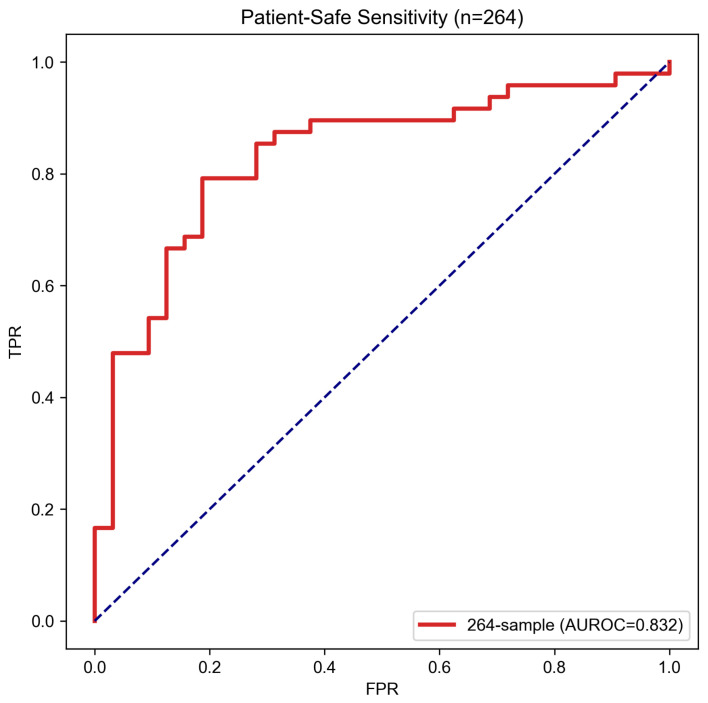
Receiver operating characteristic curve for the patient-safe sensitivity analysis on the GSE16011 cohort with four ambiguous specimens excluded (n=264; held-out test set n=80, GBM =48, LGG =32). The complete pipeline including variance filtering, ANOVA ranking, signature size selection, and Random Forest training was re-executed on the patient-safe cohort with no information from the held-out set entering any selection step. The hold-out AUROC is 0.832, virtually identical to the 0.831 obtained on the original 268-sample analysis, demonstrating that the discriminatory capacity of the signature is not attributable to any specimens with ambiguous patient identity. The red curve denotes the ROC of the 10-gene signature on the 264-sample patient-safe sensitivity cohort (AUROC = 0.832), and the blue dashed diagonal denotes the non-informative classifier reference (AUROC = 0.5).

**Figure A2:**
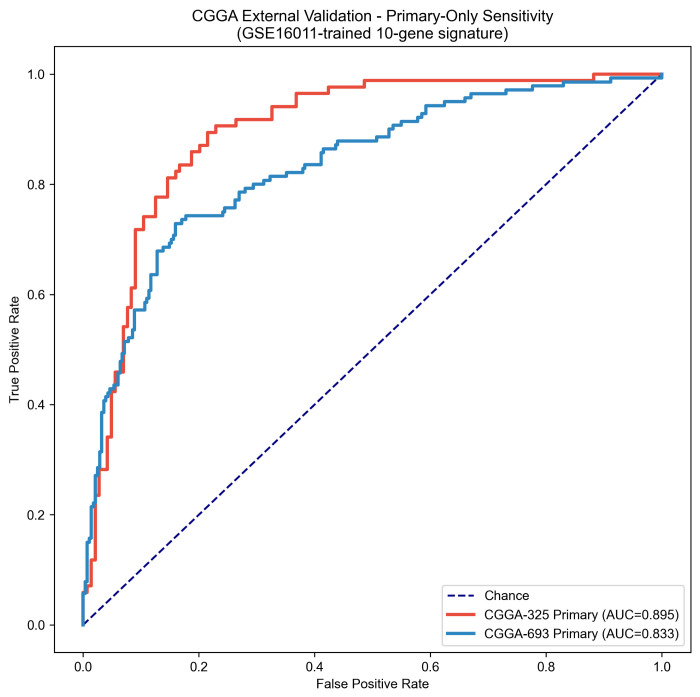
External validation receiver operating characteristic curves for the GSE16011-trained ten-gene signature applied to the Primary-only subsets of the two Chinese Glioma Genome Atlas cohorts following per-cohort z-score normalisation. CGGA-325 Primary (red, n=229; GBM =85, LGG =144) achieved AUROC =0.895, marginally exceeding the full-cohort value of 0.838. CGGA-693 Primary (blue, n=422; GBM =140, LGG =282) achieved AUROC =0.833, virtually identical to the full-cohort value of 0.836. The conservation of external classification performance after exclusion of recurrent and secondary specimens confirms that the signature does not derive its discriminatory power from longitudinal sample duplication. The red and blue curves denote the ROC of the 10-gene signature on the primary-tumour subsets of CGGA-325 (AUC = 0.895) and CGGA-693 (AUC = 0.833), and the dashed diagonal labelled “Chance” denotes the non-informative classifier reference (AUROC = 0.5).

**Figure A3:**
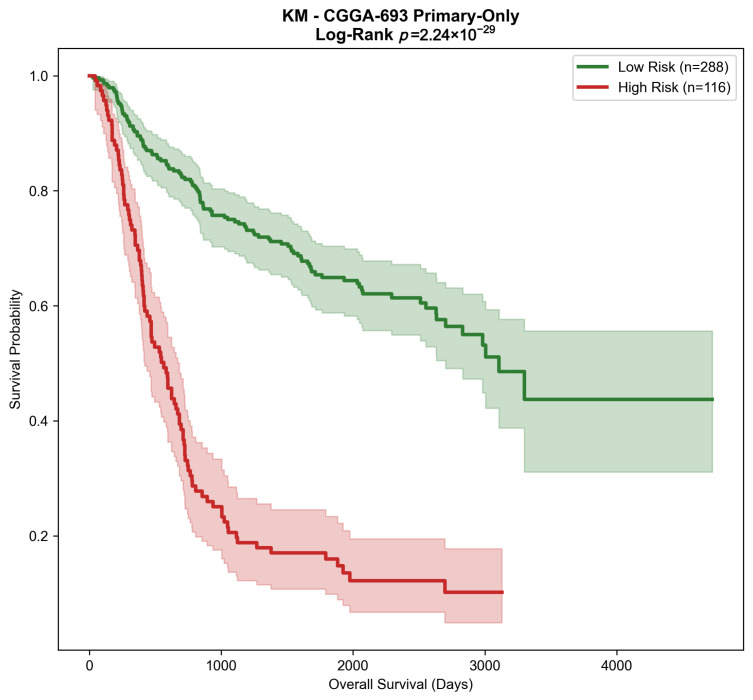
Kaplan–Meier overall survival curves in the Primary-only subset of the CGGA-693 external validation cohort (n=404; 209 events), stratified by ten-gene signature risk score using the same training-derived classification threshold applied in the main analysis. Low-risk patients (green, n=288) demonstrate significantly superior survival compared with high-risk patients (red, n=116); log-rank $p=2.24\times 10^{-29}$. Multivariate Cox proportional hazard regression in this Primary-only subset, adjusted for WHO grade, yielded a risk score hazard ratio of 9.687 (95% confidence interval: 4.452 to 21.078; p<0.001) with concordance 0.758, values equivalent to or marginally exceeding the corresponding estimates in the full mixed-PRS-type cohort. Shaded regions represent 95% confidence intervals estimated by Greenwood’s formula. The preservation of strong prognostic stratification in the Primary-only subset confirms that the survival separation reported in the main analysis is not contingent on the inclusion of recurrent specimens.

**Figure A4:**
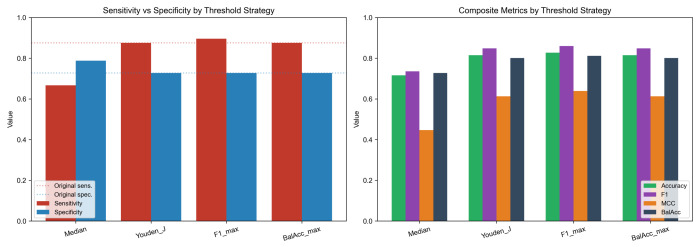
Hold-out classification metrics at four threshold selection strategies on the GSE16011 cohort (n=81). The left panel displays sensitivity (red) and specificity (blue) for each strategy, with the dashed horizontal lines indicating the values reported in the main analysis (0.875 and 0.727 respectively). The right panel displays four composite metrics: accuracy, F1-score, Matthews correlation coefficient, and balanced accuracy. The Median strategy (training set median posterior, threshold = 0.69) corresponds to the dichotomisation threshold used in the survival analysis and applies a deliberately conservative operating point that prioritises specificity over sensitivity. The Youden’s J, F1-maximising, and balanced-accuracy-maximising strategies converge on threshold values between 0.45 and 0.52 and produce mutually consistent classification performance with sensitivity between 0.875 and 0.896 and specificity 0.727. The convergence of three independent optimisation criteria within a narrow range of operating points indicates that the reported metrics are located at a stable optimum of the precision–recall trade-off surface.

**Figure A5:**
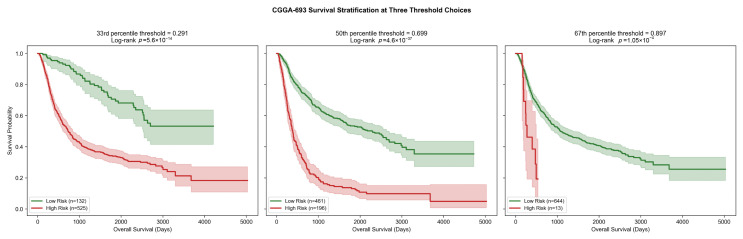
Kaplan–Meier survival curves in the CGGA-693 external validation cohort (n=657; 394 events) at three training-derived percentile thresholds applied to the predicted GBM probability distribution: 33rd percentile (left panel, threshold =0.291), 50th percentile (middle panel, threshold =0.699, used in the main analysis), and 67th percentile (right panel, threshold =0.897). All three thresholds yield highly significant log-rank separation between low-risk and high-risk groups, with *p*-values of $5.6\times 10^{-14}$, $4.6\times 10^{-37}$, and $1.0\times 10^{-4}$ respectively. The 50th percentile threshold produces the most extreme separation by virtue of the most balanced group sizes ($n_{\mathrm{low}}=461$, $n_{\mathrm{high}}=196$); the 33rd and 67th percentile thresholds produce more imbalanced splits but retain highly significant prognostic stratification, confirming that the survival result reported in the main analysis is robust to the specific dichotomisation choice. Shaded regions denote 95% confidence intervals estimated by Greenwood’s formula.

**Figure A12:**
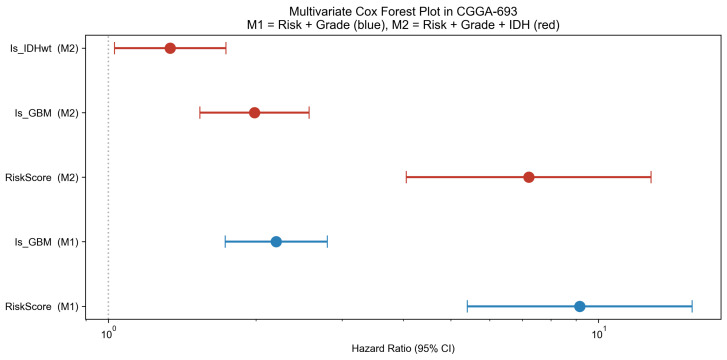
Forest plot of multivariate Cox proportional hazard regression coefficients in the CGGA-693 cohort. Blue markers correspond to model M1 (RiskScore + Grade, n=657, events =394); red markers correspond to model M2 in which IDH mutation status is added (RiskScore + Grade + IDH, n=609, events =376). Hazard ratios are plotted on a logarithmic horizontal axis with 95% confidence intervals; the dotted vertical line at HR =1 indicates the null hypothesis. The RiskScore hazard ratio attenuates from 9.16 in M1 to 7.21 in M2 but remains highly significant ($p=1.7\times 10^{-11}$), and the likelihood-ratio test between the IDH-only baseline (M3) and M2 yields $p=1.2\times 10^{-11}$, confirming the independent prognostic contribution of the risk score after adjustment for WHO grade and IDH status.

## Data Availability

The original contributions presented in the study are included in the article. Further inquiries can be directed to the corresponding authors.

## Appendix A. Overfitting Diagnostic Analyses

To characterise the extent of the overfitting and confirm that the ten-gene signature captures a genuine biological signal rather than training-specific patterns, we conducted two complementary diagnostic analyses: learning curve analysis and a label-permutation test. The results demonstrate an acceptable train-to-cross-validation gap of 8.9% and a statistically significant separation from chance performance (empirical p<0.005, 0 of 200 permutations exceeded the observed AUROC), confirming that the discriminatory performance reflects true molecular differences between GBM and LGG.

Learning curve analysis systematically varied the number of training samples from approximately 15 to 150, training a Random Forest classifier at each increment and evaluating the performance on both the training partition and a held-out cross-validation partition. The training performance remained consistently high across all the sample sizes (AUROC >0.98), while the cross-validation performance stabilised in the range from 0.89 to 0.90 from approximately 60 samples onward and showed no evidence of systematic decline at higher training fractions. The train-to-cross-validation gap at the full training set size (n=187) was 0.089, below the conventional threshold of 0.10 used to flag excessive overfitting. The stable cross-validation curve indicates that the model learns patterns generalisable to held-out data rather than memorising training-specific idiosyncrasies, and that the full training cohort size is sufficient for stable signature-level performance. The learning curve is presented in Figure 6 in the main text.

The permutation test generated a null distribution of the expected classifier performance under the hypothesis of no systematic class expression association. Training class labels (GBM versus LGG) were randomly shuffled independently for each of 200 iterations, with a complete train-predict pipeline executed for each permuted dataset. Permuted models yielded held-out AUROC values distributed near chance performance (mean =0.492, standard deviation =0.062), consistent with the expected null distribution. The true ten-gene signature achieved a held-out AUROC of 0.831, yielding an empirical value of p<0.005 (0 of 200 permuted AUROC values equalled or exceeded 0.831). The permutation test distribution is presented in Figure 7 in the main text. Collectively, the learning curve and permutation diagnostics confirm that the ten-gene signature captures a statistically robust and reproducible biological signal.

## Appendix B. Detailed Algorithmic Specifications

The complete computational pipeline integrates hypergraph construction, rough set optimisation, signature size selection, and ensemble classification through sequential algorithmic stages. This section provides detailed pseudocode and implementation specifications enabling full reproducibility.

### Appendix B.1. Hypergraph Construction Algorithm

The full procedure is summarised in Algorithm A1.

**Algorithm A1:** Hypergraph construction and hyperdegree-based gene ranking.	
**Input:**	Gene expression matrix $X\in R^{n\times p}$ where n=187 training samples
	and *p* = 12,378 probe sets after variance filtering; correlation threshold
	τ=0.75.
**Output:**	Hypergraph H=(V,E,w) with vertex set *V* (genes), hyperedge set *E*,
	and weight function *w*; ranked gene list by hyperdegree centrality.
**Procedure:**	
1. Initialise empty hyperedge set E=∅ and weight dictionary w={}.	
2. For each gene $g_{i}\in V$ (where i=1,…,p):	
(a) Compute Pearson correlation vector $r_{i}=\left[r_{i1},r_{i2},\ldots ,r_{ip}\right]$ where $r_{ij}=\mathrm{corr}\left(X_{:,i},X_{:,j}\right)$.	
(b) Define hyperedge $e_{i}=\{g_{j}:|r_{ij}|>\tau \}$.	
(c) Compute hyperedge weight $w_{e_{i}}=\sum_{g_{j}\in e_{i}}\left|r_{ij}\right|$.	
(d) Update hyperedge set: $E=E\cup \{e_{i}\}$.	
3. For each gene $g_{i}$, compute hyperdegree: $\deg_{H}\left(g_{i}\right)=\sum_{e\in E:g_{i}\in e}w_{e}$.	
4. Rank genes in descending order by $\deg_{H}\left(g_{i}\right)$.	
5. Return top 300 genes as hypergraph candidates.	

**Computational Complexity:** The algorithm requires $O(p^{2}n)$ operations for correlation matrix computation (dominant term), $O(p^{2})$ for hyperedge construction, and O(plogp) for ranking. With *p* ≈ 12,000 and n=187, the total runtime is approximately 5 to 10 min on standard hardware.

### Appendix B.2. Rough Set Reduct Optimisation Algorithm

The full procedure is summarised in Algorithm A2.

**Positive Region Computation:** For attribute subset *B*, the positive region $POS_{B}\left(D\right)$ comprises samples, x∈U, such that all the samples sharing identical attribute values in *B* belong to the same decision class. Formally, $POS_{B}\left(D\right)={⋃}_{X\in U/D}\underline{B}X$, where U/D denotes the partition of *U* by decision class, and $\underline{B}X$ denotes the *B*-lower approximation of class *X*.

**Convergence note:** The greedy heuristic converges to a locally optimal reduct but does not guarantee the globally minimal solution. Alternative reducts potentially identifiable by exhaustive search or metaheuristic optimisation are discussed in the Section 3.10.

**Algorithm A2:** Rough set reduct search via greedy forward selection.	
**Input:**	Discretised gene expression matrix $X_{\mathrm{disc}}\in {\left\{1,2,3\right\}}^{n\times 300}$ for the top
	300 hypergraph candidates; decision vector $d\in {\left\{\mathrm{GBM},\mathrm{LGG}\right\}}^{n}$.
**Output:**	Minimal reduct R⊆A achieving maximal dependency $\gamma_{R}\left(D\right)$ on the
	training set.
**Procedure:**	
1. Initialise empty reduct R=∅ and dependency $\gamma_{R}=0$.	
2. While $\gamma_{R}<1.0$:	
(a) For each gene g∈A∖R:	
i. Compute provisional attribute set $R^{\prime }=R\cup \left\{g\right\}$.	
ii. Compute positive region $POS_{R^{\prime }}\left(D\right)$ as the set of training samples whose	
decision class is unambiguously determined by attributes in $R^{\prime }$.	
iii. Compute dependency degree $\gamma_{R^{\prime }}\left(D\right)=|POS_{R^{\prime }}\left(D\right)|/|U|$.	
iv. Compute incremental gain $\Delta \gamma_{g}=\gamma_{R^{\prime }}\left(D\right)-\gamma_{R}\left(D\right)$.	
(b) Select gene with maximum gain: $g^{*}=\arg\max_{g\in A\setminus R}\Delta \gamma_{g}$.	
(c) Update reduct: $R=R\cup \{g^{*}\}$.	
(d) Update dependency: $\gamma_{R}=\gamma_{R\cup \{g^{*}\}}\left(D\right)$.	
3. Return final reduct *R* and dependency $\gamma_{R}$.	

### Appendix B.3. Signature Size Selection Procedure

Following the rough set candidate identification, the optimal signature size was determined using fivefold-stratified cross-validation on the training partition. For each candidate panel size, N∈{5,8,10,12,16,20,30,50,100}, the top *N* genes ranked using the one-way ANOVA F-statistic between GBM and LGG training samples were selected as features. A Random Forest classifier was trained and evaluated on each cross-validation fold, and the mean AUROC across five folds was recorded. The panel size maximising the mean cross-validation AUROC was selected as the final signature size (N=10, mean CV AUROC =0.906±0.029). The complete cross-validation results by panel size are reported in Table 1 in the main text.

### Appendix B.4. Discretisation Procedure

Gene expression values were discretised via equal-frequency binning to satisfy rough set requirements for categorical attributes. For each gene, $g_{j}$, in the 300-candidate set, training set expression values were divided into three bins of approximately equal sample counts:

(A1) $${\mathrm{Bin}}_{1}:x_{ij}\leq Q_{33}\left(g_{j}\right),\;{\mathrm{Bin}}_{2}:Q_{33}\left(g_{j}\right)<x_{ij}\leq Q_{67}\left(g_{j}\right),\;{\mathrm{Bin}}_{3}:x_{ij}>Q_{67}\left(g_{j}\right),$$

where $Q_{33}$ and $Q_{67}$ denote the 33rd and 67th percentiles of the training set expression for gene $g_{j}$. Bin boundaries computed in the training set were applied consistently to all the validation and test partitions to prevent information leakage. Sensitivity analyses using equal-width binning and entropy-based discretisation yielded similar reduct sizes and overlapping gene memberships, indicating robustness to the discretisation strategy.

## Appendix C. Hypergraph Theory Mathematical Framework

A hypergraph, H=(V,E), generalises classical graphs by permitting hyperedges, e∈E, to connect arbitrary subsets of vertices rather than restricting edges to vertex pairs. Formally, *V* is a finite vertex set, and $E\subseteq 2^{V}\setminus \left\{\emptyset \right\}$ is a family of nonempty subsets of *V* called hyperedges. A weighted hypergraph additionally assigns weights, $w:E\to R^{+}$, to each hyperedge.

In biological applications, hypergraphs naturally represent higher-order interactions, such as protein complexes, metabolic reactions, and co-regulated gene modules exhibiting correlated expressions. This contrasts with pairwise graphs, which cannot directly encode multi-way relationships without auxiliary constructions.

### Appendix C.1. Hyperdegree Centrality

The hyperdegree of vertex v∈V quantifies its topological participation across hyperedges. For unweighted hypergraphs, $\deg_{H}\left(v\right)=\left|\left\{e\in E:v\in e\right\}\right|$ counts the number of hyperedges containing *v*. For weighted hypergraphs, the generalised hyperdegree aggregates edge weights:

(A2) $$\deg_{H}\left(v\right)=\sum_{e\in E:v\in e}w\left(e\right).$$

Vertices with high hyperdegrees are topological hubs participating in many co-expression modules, potentially serving as regulatory integration points across multiple biological pathways.

### Appendix C.2. Threshold Selection Rationale

The correlation threshold τ=0.75 was selected to balance the hypergraph density and biological specificity. Thresholds ranging from 0.60 to 0.90 in increments of 0.05 were evaluated based on two criteria: maximisation of subsequent rough set reduct discriminatory performance in cross-validation and biological interpretability assessed via the Gene Ontology enrichment of top-ranked genes. The threshold τ=0.75 satisfied both criteria, and alternative thresholds in the range from 0.70 to 0.80 yielded similar candidate gene sets with substantial overlap (>80% gene membership in common), indicating robustness to the specific threshold value.

## Appendix D. Rough Set Theory Mathematical Framework

Rough set theory provides a mathematical framework for knowledge discovery under uncertainty and incompleteness. The fundamental concept is the approximation of arbitrary object subsets using equivalence classes induced by available attributes.

### Appendix D.1. Information Systems and Indiscernibility

An information system is a tuple S=(U,A) where *U* is a nonempty finite set of objects (samples) and *A* is a nonempty finite set of attributes (features). Each attribute a∈A defines a function $a:U\to V_{a}$ mapping objects to attribute values in domain $V_{a}$. A decision system extends this as S=(U,A∪{d}) where d∉A is a distinguished decision attribute.

For attribute subset B⊆A, the indiscernibility relation IND(B) is an equivalence relation on *U* defined by:

(A3) IND(B)={(x,y)∈U×U:∀a∈B,a(x)=a(y)}.

Two objects are indiscernible with respect to *B* if they possess identical values for all the attributes in *B*. The equivalence classes of IND(B) partition *U* into blocks ${\left[x\right]}_{B}=\left\{y\in U:\left(x,y\right)\in IND\left(B\right)\right\}$ of objects indistinguishable using attributes in *B*.

### Appendix D.2. Lower and Upper Approximations

For arbitrary subset X⊆U, the *B*-lower approximation $\underline{B}X$ comprises objects certainly belonging to *X* based on knowledge in *B*:

(A4) $$\underline{B}X=\left\{x\in U:{\left[x\right]}_{B}\subseteq X\right\}.$$

The *B*-upper approximation $\bar{B}X$ comprises objects possibly belonging to *X*:

(A5) $$\bar{B}X=\left\{x\in U:{\left[x\right]}_{B}\cap X\neq \emptyset \right\}.$$

The boundary region $BN_{B}\left(X\right)=\bar{B}X\setminus \underline{B}X$ contains objects that cannot be classified with certainty.

### Appendix D.3. Positive Region and Dependency Degree

For decision attribute *d* inducing partition $U/d=\{D_{1},D_{2},\ldots ,D_{k}\}$, the positive region of *d* with respect to *B* is:

(A6) $$POS_{B}\left(d\right)=\overset{k}{\underset{i=1}{⋃}}\underline{B}D_{i}.$$

This comprises objects whose decision class can be deterministically inferred from attributes in *B*. The dependency degree quantifies the proportion of classifiable objects:

(A7) $$\gamma_{B}\left(d\right)=\frac{|POS_{B}\left(d\right)|}{\left|U\right|},$$

where $0\leq \gamma_{B}\left(d\right)\leq 1$. Full dependency $\gamma_{B}\left(d\right)=1$ indicates perfect classification power of attribute set *B* over the decision attribute *d*.

### Appendix D.4. Reduct Definition and Properties

An attribute subset R⊆A is a reduct if it satisfies two conditions:

1. $\gamma_{R}\left(d\right)=\gamma_{A}\left(d\right)$ (preservation: *R* maintains the full classification power of *A*)
2. $\forall r\in R,\;\gamma_{R\setminus \{r\}}\left(d\right)<\gamma_{R}\left(d\right)$ (minimality: no attribute in *R* is redundant)

A reduct represents a minimal sufficient attribute set for classification. An information system may possess multiple distinct reducts achieving the same dependency degree, reflecting alternative minimal explanations of the decision attribute. The intersection of all the reducts, referred to as the core, comprises indispensable attributes appearing in every reduct.

## Appendix E. Random Forest Hyperparameter Optimisation

Random Forest hyperparameters were selected via nested cross-validation to prevent information leakage and overfitting. The hyperparameter grid comprised number of trees {50,100,200}, maximum tree depth {5,10,15,None}, minimum samples per leaf {1,2,3,5}, and maximum features per split $\left\{\sqrt{p},\log_{2}\left(p\right),p\right\}$ where p=10 denotes the final signature size. Each combination was evaluated via fivefold cross-validation on the training partition, with performance quantified by mean AUROC across folds.

The optimal configuration comprised 200 trees, maximum tree depth 10, minimum samples per leaf 3, class_weight set to “balanced”, and maximum features $\lfloor \sqrt{10}\rfloor =3$ per split. Increasing tree count from 100 to 200 yielded a consistent marginal improvement in mean CV AUROC (approximately 0.003), and no further improvement was observed at 500 trees. Maximum depth of 10 prevented terminal node splitting to individual samples while allowing sufficient tree complexity to capture feature interactions. Minimum samples per leaf of 3 prevented terminal nodes from fitting individual sample noise, which is particularly relevant given the modest training cohort size of n=187. The balanced class weight compensates for the 111:76 GBM-to-LGG training imbalance by scaling the contribution of minority-class samples in the impurity criterion, improving sensitivity for LGG classification without manual resampling. Maximum features of 3 per split introduced sufficient randomness for ensemble diversity while maintaining individual tree discriminative performance.

## Appendix F. Patient-Level Sensitivity Analysis Figures

This appendix presents the receiver operating characteristic and Kaplan–Meier curves corresponding to the three patient-level sensitivity analyses described in Section 2.15 of the main text. The underlying numerical results are summarised in the side-by-side comparison table embedded within that section. The first analysis re-executes the complete feature selection and classification pipeline on the GSE16011 cohort after excluding four specimens flagged in Section 4.5 as potentially representing same-patient malignant progression. The second analysis applies the frozen ten-gene signature trained on the original 268-sample cohort to the CGGA external validation cohorts restricted to specimens annotated as PRS_type == Primary, eliminating any longitudinal recurrent samples that could share patient identity with primary specimens elsewhere in the same cohort. The third analysis repeats the Kaplan–Meier prognostic stratification in the CGGA-693 Primary subset to confirm that the survival separation reported in the main analysis is not contingent on the inclusion of recurrent samples.

## Appendix G. Threshold Optimisation and Survival Optimism Figures

This appendix presents the supporting figures for the threshold optimisation sensitivity analysis described in Section 2.4 and the survival analysis robustness assessment described in Section 2.14 of the main text. The figures complement the numerical comparisons summarised in Table 3 and Table 9 by providing visual confirmation that the classification operating point and the survival prognostic stratification are stable across reasonable methodological perturbations.

## Appendix H. Validation Extras Figures

This appendix presents the supporting figures for the four validation analyses described in Section 2.5, Section 2.9, Section 2.10 and Section 4.8 of the main text. The figures complement the numerical comparisons summarised in Table 5 and Table 6 and provide visual confirmation that the hyperedge correlation threshold, the gene selection pipeline, the cross-cohort discrimination performance, and the absolute probability calibration each behave as described in the corresponding main-text subsections.

## Appendix I. IDH Stratification and Baseline Comparison Figures

This appendix presents the supporting figures for the four analyses concerning the relationship of the 10-gene signature to established clinical and molecular markers, described in Section 2.8, Section 2.9, Section 2.10, Section 2.11, Section 2.12, Section 2.13, Section 2.14, Section 2.15, Section 2.16 and Section 2.17 of the main text.

## Appendix J. Reduct Stability, Leave-One-Out, and Normalisation Figures

This appendix presents the supporting figures for the three analyses concerning gene-set robustness and clinical translation described in Section 2.18, Section 2.19 and Section 4.4 of the main text.

## References

[1] Pellerino A., Caccese M., Padovan M., Cerretti G., Lombardi G. (2022). Epidemiology, risk factors, and prognostic factors of gliomas. Clin. Transl. Imaging.

[2] Grochans S., Cybulska A.M., Simińska D., Korbecki J., Kojder K., Chlubek D., Baranowska-Bosiacka I. (2022). Epidemiology of glioblastoma multiforme–literature review. Cancers.

[3] Hua W., Zhang X. (2024). Clinical Trials on Glioma. Progress in the Diagnosis and Treatment of Gliomas.

[4] Oster C., Schmidt T., Agkatsev S., Lazaridis L., Kleinschnitz C., Sure U., Scheffler B., Kebir S., Glas M. (2023). Are we providing best-available care to newly diagnosed glioblastoma patients? Systematic review of phase III trials in newly diagnosed glioblastoma 2005–2022. Neuro-Oncol. Adv..

[5] Lai S., Li P., Liu X., Liu G., Xie T., Zhang X., Wang X., Huang J., Tang Y., Liu Z. (2024). Efficacy and safety of anlotinib combined with the STUPP regimen in patients with newly diagnosed glioblastoma: A multicenter, single-arm, phase II trial. Cancer Biol. Med..

[6] McMahon D., Gleeson J., O’reilly S., Bambury R. (2022). Management of newly diagnosed glioblastoma multiforme: Current state of the art and emerging therapeutic approaches. Med. Oncol..

[7] Louis D.N., Perry A., Wesseling P., Brat D.J., Cree I.A., Figarella-Branger D., Hawkins C., Ng H., Pfister S.M., Reifenberger G. (2021). The 2021 WHO classification of tumors of the central nervous system: A summary. Neuro-Oncol..

[8] Noushmehr H., Weisenberger D.J., Diefes K., Phillips H.S., Pujara K., Berman B.P., Pan F., Pelloski C.E., Sulman E.P., Bhat K.P. (2010). Identification of a CpG island methylator phenotype that defines a distinct subgroup of glioma. Cancer Cell.

[9] Paweł K., Maria Małgorzata S. (2022). CpG island methylator phenotype—a hope for the future or a road to nowhere?. Int. J. Mol. Sci..

[10] Gibson D., Vo A.H., Lambing H., Bhattacharya P., Tahir P., Chehab F.F., Butowski N. (2024). A systematic review of high impact CpG sites and regions for MGMT methylation in glioblastoma [A systematic review of MGMT methylation in GBM]. BMC Neurol..

[11] Eisenbarth D., Wang Y.A. (2023). Glioblastoma heterogeneity at single cell resolution. Oncogene.

[12] Garcia-Montano L.A., Licon-Munoz Y., Martinez F.J., Keddari Y.R., Ziemke M.K., Chohan M.O., Piccirillo S.G. (2023). Dissecting intra-tumor heterogeneity in the glioblastoma microenvironment using fluorescence-guided multiple sampling. Mol. Cancer Res..

[13] Wu H., Guo C., Wang C., Xu J., Zheng S., Duan J., Li Y., Bai H., Xu Q., Ning F. (2023). Single-cell RNA sequencing reveals tumor heterogeneity, microenvironment, and drug-resistance mechanisms of recurrent glioblastoma. Cancer Sci..

[14] Capper D., Reifenberger G., French P.J., Schweizer L., Weller M., Touat M., Niclou S.P., Euskirchen P., Haberler C., Hegi M.E. (2023). EANO guideline on rational molecular testing of gliomas, glioneuronal, and neuronal tumors in adults for targeted therapy selection. Neuro-Oncol..

[15] Brandner S., Von Deimling A. (2015). Diagnostic, prognostic and predictive relevance of molecular markers in gliomas. Neuropathol. Appl. Neurobiol..

[16] Abou Tayoun A.N., Burchard P.R., Malik I., Scherer A., Tsongalis G.J. (2014). Democratizing molecular diagnostics for the developing world. Am. J. Clin. Pathol..

[17] Chakraborty S. (2024). Democratizing nucleic acid-based molecular diagnostic tests for infectious diseases at resource-limited settings–from point of care to extreme point of care. Sens. Diagn..

[18] Shi J. (2022). Machine learning and bioinformatics approaches for classification and clinical detection of bevacizumab responsive glioblastoma subtypes based on miRNA expression. Sci. Rep..

[19] Yang P., Feng P., Tian G., Zhao G., Yuan G., Pan Y. (2025). Integrative machine learning and bioinformatics analysis unveil key genes for precise glioma classification and prognosis evaluation. Comput. Biol. Chem..

[20] Bhatele K.R., Bhadauria S.S. (2022). Machine learning application in glioma classification: Review and comparison analysis. Arch. Comput. Methods Eng..

[21] Osmak G., Pisklova M. (2025). Transcriptomics and the “curse of dimensionality”: Monte Carlo simulations of ml-models as a tool for analyzing multidimensional data in tasks of searching markers of biological processes. Mol. Biol..

[22] Al-Azani S., Alkhnbashi O.S., Ramadan E., Alfarraj M. (2024). Gene expression-based cancer classification for handling the class imbalance problem and curse of dimensionality. Int. J. Mol. Sci..

[23] Imoto Y., Nakamura T., Escolar E.G., Yoshiwaki M., Kojima Y., Yabuta Y., Katou Y., Yamamoto T., Hiraoka Y., Saitou M. (2022). Resolution of the curse of dimensionality in single-cell RNA sequencing data analysis. Life Sci. Alliance.

[24] Zitnik M., Li M.M., Wells A., Glass K., Morselli Gysi D., Krishnan A., Murali T., Radivojac P., Roy S., Baudot A. (2024). Current and future directions in network biology. Bioinform. Adv..

[25] Sultan S.E., Moczek A.P., Walsh D. (2022). Bridging the explanatory gaps: What can we learn from a biological agency perspective?. BioEssays.

[26] Zhang P., Zhang D., Zhou W., Wang L., Wang B., Zhang T., Li S. (2024). Network pharmacology: Towards the artificial intelligence-based precision traditional Chinese medicine. Brief. Bioinform..

[27] Cen G., Liu L., Wang J., Wang X., Chen S., Song Y., Liang Z. (2022). Weighted Gene Co-Expression Network Analysis to Identify Potential Biological Processes and Key Genes in COVID-19-Related Stroke. Oxidative Med. Cell. Longev..

[28] Johnson K.A., Krishnan A. (2022). Robust normalization and transformation techniques for constructing gene coexpression networks from RNA-seq data. Genome Biol..

[29] Zhang S., Jiang C., Jiang L., Chen H., Huang J., Gao X., Xia Z., Tran L.J., Zhang J., Chi H. (2023). Construction of a diagnostic model for hepatitis B-related hepatocellular carcinoma using machine learning and artificial neural networks and revealing the correlation by immunoassay. Tumour Virus Res..

[30] Watson D.S. (2022). Interpretable machine learning for genomics. Hum. Genet..

[31] Chen V., Yang M., Cui W., Kim J.S., Talwalkar A., Ma J. (2024). Applying interpretable machine learning in computational biology—pitfalls, recommendations and opportunities for new developments. Nat. Methods.

[32] Bretto A. (2013). Hypergraph theory. An Introduction. Mathematical Engineering.

[33] Feng S., Heath E., Jefferson B., Joslyn C., Kvinge H., Mitchell H.D., Praggastis B., Eisfeld A.J., Sims A.C., Thackray L.B. (2021). Hypergraph models of biological networks to identify genes critical to pathogenic viral response. BMC Bioinform..

[34] Franzese N., Groce A., Murali T., Ritz A. (2019). Hypergraph-based connectivity measures for signaling pathway topologies. PLoS Comput. Biol..

[35] Kong Y., Yu T. (2019). A hypergraph-based method for large-scale dynamic correlation study at the transcriptomic scale. BMC Genom..

[36] Estrada E., Rodríguez-Velázquez J.A. (2006). Subgraph centrality and clustering in complex hyper-networks. Phys. A Stat. Mech. Its Appl..

[37] law Pawlak Z. (1982). Rough sets. Int. J. Comput. Inf. Sci..

[38] Swiniarski R.W., Skowron A. (2003). Rough set methods in feature selection and recognition. Pattern Recognit. Lett..

[39] Abu Khurma R., Aljarah I., Sharieh A., Abd Elaziz M., Damaševičius R., Krilavičius T. (2022). A review of the modification strategies of the nature inspired algorithms for feature selection problem. Mathematics.

[40] Gravendeel L.A., Kouwenhoven M.C., Gevaert O., de Rooi J.J., Stubbs A.P., Duijm J.E., Daemen A., Bleeker F.E., Bralten L.B., Kloosterhof N.K. (2009). Intrinsic gene expression profiles of gliomas are a better predictor of survival than histology. Cancer Res..

[41] Boisen M.K., Holst C.B., Consalvo N., Chinot O.L., Johansen J.S. (2017). Plasma YKL-40 as a biomarker for bevacizumab efficacy in patients with newly diagnosed glioblastoma in the phase 3 randomized AVAglio trial. Oncotarget.

[42] Qin G., Li X., Chen Z., Liao G., Su Y., Chen Y., Zhang W. (2017). Prognostic value of YKL-40 in patients with glioblastoma: A systematic review and meta-analysis. Mol. Neurobiol..

[43] Wolf A., Agnihotri S., Micallef J., Mukherjee J., Sabha N., Cairns R., Hawkins C., Guha A. (2011). Hexokinase 2 is a key mediator of aerobic glycolysis and promotes tumor growth in human glioblastoma multiforme. J. Exp. Med..

[44] Li H., Yang W., Wang S., Zhao Z., Wang W., Shi M., Li Y. (2025). Adrenomedullin in tumorigenesis and cancer progression. Int. J. Mol. Sci..

[45] Song X., Zhu Q., Zhang J., Yang J., Zhang X., Song Q. (2025). Trigeminal nerve-driven neurogenic inflammation linking migraine to glioblastoma invasion: A literature review. Front. Immunol..

[46] Zheng Y., Jiang H., Yang N., Shen S., Huang D., Jia L., Ling J., Xu L., Li M., Yu K. (2024). Glioma-derived ANXA1 suppresses the immune response to TLR3 ligands by promoting an anti-inflammatory tumor microenvironment. Cell. Mol. Immunol..

[47] Gao K., Li X., Luo S., Zhao L. (2024). An overview of the regulatory role of annexin A1 in the tumor microenvironment and its prospective clinical application. Int. J. Oncol..

[48] Tuysuz E.C., Mourati E., Rosberg R., Moskal A., Gialeli C., Johansson E., Governa V., Belting M., Pietras A., Blom A.M. (2024). Tumor suppressor role of the complement inhibitor CSMD1 and its role in TNF-induced neuroinflammation in gliomas. J. Exp. Clin. Cancer Res..

[49] Verhaak R.G., Hoadley K.A., Purdom E., Wang V., Qi Y., Wilkerson M.D., Miller C.R., Ding L., Golub T., Mesirov J.P. (2010). Integrated genomic analysis identifies clinically relevant subtypes of glioblastoma characterized by abnormalities in PDGFRA, IDH1, EGFR, and NF1. Cancer Cell.

[50] You J.E., Jung S.H., Kim P.H. (2021). The effect of annexin A1 as a potential new therapeutic target on neuronal damage by activated microglia. Mol. Cells.

